# Unveiling adolescents’ climate agency modes and their personal, behavioral, and environmental determinants: a qualitative study

**DOI:** 10.3389/fpsyg.2026.1882370

**Published:** 2026-09-17

**Authors:** Jennifer Cunha, Rafaela Carneiro, Marcelo Félix, Pedro Rosário

**Affiliations:** Psychology Research Center, School of Psychology, University of Minho, Braga, Portugal

**Keywords:** adolescents, climate agency, climate change, human agency, knowledge, mitigation behaviors

## Abstract

Climate change severely affects the planet and individuals’ health and well-being. To take urgent action to mitigate this phenomenon, literature has advocated that adolescents can be active agents and protagonists of change, but how are they responding to these challenges? Grounded in Bandura’s theory of agency and the triadic reciprocal causation model, this study explored adolescents’ agency modes toward climate change and perceived personal, behavioral, and environmental determinants. Participants were 51 adolescents enrolled in middle and high schools. Data collected through semi-structured interviews with photo-elicitation and a focus group discussion were analyzed using content analysis. Findings revealed that adolescents at both school levels mainly report engaging in individual and collective actions to address climate change that do not simultaneously reflect the properties of human agency. Personal (e.g., superficial knowledge, low expected outcomes), behavioral (e.g., lack of control), and environmental (e.g., lack of community resources) determinants may help explain the current findings. Salient differences in data patterns among middle and high school students were reported and discussed. This study provides implications for future research and prompts reflection on educational practice.

## 1 Introduction

Climate change (CC) is an urgent and irreversible threat to current and future society (Sanson et al., 2019). According to the Intergovernmental Panel on Climate Change (Intergovernmental Panel on Climate Change, 2023), CC refers to long-term shifts in Earth’s weather and climate patterns (e.g., temperature, sea level, precipitation), primarily driven by increased concentrations of greenhouse gases derived from human activities. In this scenario, policy-level measures alongside citizens’ individual and collective actions to reduce greenhouse gas emissions (Intergovernmental Panel on Climate Change, 2023) are key. Integrated responses from all levels of society are urgent, as CC can cause droughts, wildfires, sea level rise, floods, and altered crop-growing seasons, likely resulting in illness, injury, malnutrition, and death (Garcia and Sheehan, 2016; Intergovernmental Panel on Climate Change, 2023). Children and adolescents are among the age groups most susceptible to the proximal and long-term impacts of this phenomenon (e.g., CC-induced disasters and resource shortages; Corner et al., 2015; Garcia and Sheehan, 2016; Han and Ahn, 2020). Following this reasoning, Sanson et al. (2019) alert that children and youth can be proactive agents in the fight against CC. However, research is still scarce on how adolescents exercise their agency to address CC (Koskela and Paloniemi, 2023), henceforth termed “climate agency.” The current study addresses this gap while mapping the personal, behavioral, and environmental determinants of adolescents’ climate agency specific to mitigation actions.

### 1.1 Theoretical framework

As CC has become an increasingly pressing global challenge, numerous theoretical frameworks have been used to understand the determinants of pro-environmental behavior. For instance, Ajzen’s (2012) Theory of Planned Behavior has been influential in the field for highlighting how attitudes, subjective norms, and perceived control indirectly predict pro-environmental behavior. A study on climate-friendly food choices among adolescents (Jylhä et al., 2023) extended this model by highlighting the added role of emotions, particularly CC worry. However, by design, this theory focuses on behavioral intentions, and substantial literature documents the gap between intentions and actual behaviors (e.g., Colombo et al., 2023b). In addressing antecedents of pro-environmental behavior, the triple-A framework (Hamann et al., 2024) advances the theory on self-efficacy by distinguishing efficacy beliefs about the agent, the action, and the aims. Van Valkengoed and Steg’s (2024) Climate Anxiety Compass also zooms in to map how different coping strategies in response to climate anxiety can lead to individual or collective actions. For a deeper perspective on collective action, the Social Identity Model of Pro-Environmental Action (Fritsche et al., 2018) shows how ingroup identification, perceived collective efficacy, and ingroup norms and goals give rise to collective emotions and motivations that shape environmental action.

Together, these frameworks illustrate several relevant variables that shape human engagement in CC mitigation behavior. However, the scale and systemic change needed to address CC call for a more integrative lens on how individuals and groups exercise intentional control over their actions and the environment, i.e., agency (Koskela and Paloniemi, 2023). This concept has been examined from diverse theoretical perspectives. For instance, the ecological (Priestley et al., 2012) and performative (Firinci Orman, 2022) perspectives conceptualize agency as an emergent and context-dependent phenomenon, shaped by individuals’ capacity to act within and against social norms and material constraints. However, while also addressing the latter, the human agency theory (Bandura, 1999) explicitly emphasizes self-regulatory skills as core components through which people become and remain active agents in response to a global problem such as CC. Following this reasoning, Bandura’s social cognitive theory (2006), particularly human agency theory and the triadic reciprocal causation model, grounds the current study.

This theoretical framework presents three distinct modes of agency: individual, collective, and proxy. Individual agency comprises the exercise of personal influence over one’s functioning and surroundings; collective agency results from shared beliefs and the ability of groups of people to work together toward a goal. These efforts involve people’s knowledge, skills, and resources, enabling them to act together and shape the future (Bandura, 2006). Finally, proxy agency refers to the influence of people acting on behalf of those without direct control over the conditions affecting their lives (Bandura, 2006). This framework states that human agency (concretely, each of the modes) encompasses three properties: forethought, self-reactiveness, and self-reflectiveness (Bandura, 2018). Forethought involves motivation to achieve the future intended; namely, it describes individuals’ efforts to create action plans, set goals, and visualize desired outcomes. Self-reactiveness refers to individuals’ ability to self-regulate behavior, manage actions, and evaluate their performance toward self-set goals. Finally, self-reflectiveness involves reflection on values, effectiveness in confronting challenges, and consistency between thoughts and actions.

According to the triadic reciprocal causation model, the modes of agency are influenced by the reciprocal interaction between personal, behavioral, and environmental determinants (Bandura, 1999, 2018). Personal determinants include biological, cognitive, affective, and motivational variables (e.g., self-efficacy, outcome expectations, goals, and self-evaluation) (Bandura, 1999). They interact with behavioral determinants (e.g., choices people make, how they perform, and manage their lives; Usher and Ford, 2022), which play an important role in producing events, as Bandura (1986) noted. Finally, environmental determinants influence human functioning. These determinants include physical and social surroundings, peers, groups, and family (Usher and Ford, 2022), interacting to shape human behavior. Following a reciprocal interaction mode, environmental determinants are likely to impact cognitive and motivational variables, while individual actions influence the surrounding context.

### 1.2 Personal determinants and adolescents’ behavior toward CC

Literature has reported personal determinants likely to contribute positively or negatively to young people’s CC mitigation behavior. For example, knowledge (Chang and Pascua, 2016), self-efficacy (Du et al., 2023), and outcome expectations (Corner et al., 2015).

Prior research indicates that adolescents’ perceptions and knowledge of CC are limited (e.g., Southeast Asia: Chang and Pascua, 2016; Karpudewan et al., 2015; West Asia: Doğru and Yüzbaşıoğlu, 2023; Özcan Tan and Demirkaya, 2025). For example, a study in North America and Western Europe by Thomas et al. (2022) with participants aged 7–18 indicated that younger participants associated CC with concrete environmental changes, such as melting the polar ice caps or losing animal species. No information was reported about older participants, who considered that CC was insufficiently covered in class. Consistently, Southeast Asia research (e.g., Chang and Pascua, 2016; Karpudewan et al., 2015) reports that the CC-related concepts covered in the curriculum are often broad and ill-defined, preventing adolescents from recognizing and relating them correctly. For example, Chang and Pascua (2016) found that some students linked transport and electricity to CC, but their explanations were unrelated to fossil fuel use or carbon emissions. These results may help understand data from a North American study (Busch et al., 2019) that found a weak relationship between CC knowledge and youth CC mitigation behaviors.

As literature reports, self-efficacy and outcome expectations are also determinants of CC mitigation behaviors among children and adolescents (Baldwin et al., 2023; Buttigieg and Pace, 2013; Corner et al., 2015). Bandura (1986) defines self-efficacy as the individual’s judgments of their ability to organize and execute courses of action necessary to achieve goals at a particular level. However, it is important to note that collective efficacy gains particular relevance in a global environmental problem such as CC, as adolescents may feel powerless to act alone (Bandura and Cherry, 2020; Ojala, 2023). For example, Corner et al. (2015) reported that many adolescents perceive that their individual actions do not significantly impact the mitigation of a global environmental issue such as CC. This perception may be related to adolescents’ outcome expectancy, which refers to individuals’ judgments of a behavior’s likely consequences or results (Bandura, 1986). According to Shell (2023), outcome expectancies derived from successful past experiences lead individuals to anticipate subsequent outcomes. This is particularly relevant because, as Buttigieg and Pace (2013) found, adolescents with low self-efficacy regarding CC also have little direct experience in mitigating CC, which may limit their ability to visualize the results of their actions and lead to lower outcome expectancy (Shell, 2023).

In addition, prior studies report that adolescents tend to attribute the responsibility for the responses to CC to others (e.g., governments) while underestimating their responsibility (Corner et al., 2015; Oliveira et al., 2015). For example, García-Vinuesa et al. (2021) reported a lack of personal responsibility related to CC among Portuguese adolescents that was likely to hinder pro-environmental behavior.

Research also indicates that despite reporting being very interested and concerned about CC, adolescents often prioritize other activities (e.g., leisure activities) (Corner et al., 2015), and their concern about CC seems to decrease when considering personal benefits or immediate tasks (i.e., homework) (Line et al., 2010). However, some adolescents maintain, or even increase, concern and CC mitigation behaviors regardless of other events in their lives or of the support from their family or friends (Brown and Krettenauer, 2025; Sanson et al., 2019). The latter findings are particularly relevant because CC concern positively predicts adolescents’ involvement with CC (Ojala, 2023).

Adolescents typically address CC by engaging in various actions such as recycling, reusing materials, saving energy, and using public transport (Brown and Krettenauer, 2025; McNeill and Vaughn, 2012; Pickering et al., 2021; Tasti and Akar, 2021). However, only a few report high-impact behaviors such as reducing meat consumption or not using a private car (Pickering et al., 2021). These data may help explain researchers’ emphasis on exposing adolescents to behaviors with high environmental impact and facilitating the integration of CC mitigation behaviors into their daily lives (Colombo et al., 2023a; Pickering et al., 2020). Addressing perceived barriers and making these actions more accessible are efforts likely to promote a deeper and more sustained involvement of young people in combating CC (Baldwin et al., 2023). Importantly, adolescents can also inform others (e.g., peers, family) about CC while persuading them to engage in CC mitigation behaviors and influence the local or central political representatives on CC policies, for example, by writing letters (Baldwin et al., 2023; Žukauskienė et al., 2021).

In addition, adolescents participate in beach and school clean-ups, enrollment in protests demanding more effective action from governments and companies, and in awareness campaigns about the climate crisis or school climate strikes (Baldwin et al., 2023; Corner et al., 2015; Han and Ahn, 2020). For instance, while climate strikes help expand people’s CC awareness worldwide, adolescents still sense limited power over immediate macro changes (Han and Ahn, 2020). This may help explain data showing that collective protest is related to negative affect and even psychological distress (Curll et al., 2022; Ediz and Yanik, 2023).

### 1.3 Environmental determinants and adolescents’ behavior toward CC

The promotion of educational environments likely to engage adolescents in experiential activities and encourage civic engagement is key to empowering the next generations to face this global challenge effectively and resiliently (Corner et al., 2015; Sanson et al., 2019). Specifically, the role of micro and macro systems (i.e., formal and non-formal educational settings, family, and media) has been studied. Despite CC education being key to promoting youth climate agency (UNICEF, 2019), Rousell and Cutter-Mackenzie-Knowles (2020) concluded in their systematic review that it has been ineffective in changing behaviors, as programs were mainly focused on delivering scientific knowledge. Moreover, other studies (e.g., Denault et al., 2024; Valdez et al., 2018; Wu and Otsuka, 2021) suggest that discussions with teachers about CC do not seem to influence adolescents’ pro-environmental behavior. However, Monroe et al. (2017) found that efforts beyond the formal classroom, such as school (e.g., collecting and monitoring energy use) or community service projects, are beneficial in instigating CC mitigation behavior. In addition, Hohenhaus et al. (2023) concluded that CC interventions that foster both knowledge and self-efficacy, combined with at least one other component (e.g., goal setting, social environment, such as peer interactions, or nature connectedness), are more likely to lead to behavior change. For example, adolescents’ participation in ecological activities or environmental clubs is associated with increased pro-environmental behavior (Wu and Otsuka, 2021). Moreover, Flora et al. (2014) indicated that high school students from the USA who participated in an “education and entertainment” assembly focused on CC showed better scientific knowledge, more pro-environmental attitudes, and pro-environmental behaviors when comparing pre- and post-assembly means. In addition, these activities also encouraged informal learning through interpersonal discussions with parents and friends, which is crucial for forming and reinforcing new knowledge and behaviors. However, it is important to note that social and economic inequalities shape access to and quality of formal, informal, and non-formal CC educational opportunities, which may further limit adolescents’ responses to CC (UNICEF, 2019).

The family context plays a key role in adolescents’ development; however, despite its importance, prior studies have shown inconsistent results regarding the relationships between parents’ and their children’s attitudes, beliefs, and behaviors related to CC. While studies have shown a positive association between parents’ pro-environmental attitudes and behaviors and adolescents’ pro-environmental behaviors, other studies have reported non-significant results (Collado et al., 2019; Denault et al., 2024; Evans et al., 2018; Shrum et al., 2023). The study by Lawson et al. (2019) adds that family discussion about CC is a stronger predictor of children’s reported climate behaviors than parents’ climate behaviors. Consistently, Baker et al. (2021) found that, despite adults (parents and teachers) reporting their own anxiety about CC as the main barrier to conversations about the topic, participants also reported positive experiences, such as noticing behavioral changes in the child’s personal actions. On the other hand, the family context is not always a facilitator, as some adolescents reported family-related barriers to adopting a more sustainable lifestyle (e.g., family meat consumption habits) (Thomas et al., 2022). Taken together, the family’s role in adolescents’ pro-environmental behavior depends mainly on how parental attitudes are conveyed to and perceived by young people.

Lastly, media and social networks are important and frequent sources of information about CC, especially for adolescents (Wu and Otsuka, 2021). However, studies conducted with adolescents found that mass and social media exposure and engagement are not significantly related to adolescents’ pro-environmental behaviors (Gonzalez et al., 2023; Wu and Otsuka, 2021). The study by Brown and Krettenauer (2025) may help understand these findings. While pro-environmentally engaged adolescents reported actively using social media to deepen their knowledge and translate it into action, disengagers struggled to connect emotionally with the issue and approached information superficially. As a prior study showed, exposure to CC content on social media does not necessarily guarantee direct engagement among adolescents; the importance and attention given to the CC content are crucial (Rousseau, 2024).

In sum, extant literature stresses the relevant and complex role of distinct contexts, such as formal and non-formal education, family, or the media, in educating and promoting adolescents’ CC mitigation behavior (Baldwin et al., 2023; Corner et al., 2015; Hohenhaus et al., 2023; Thomas et al., 2022). However, a human agency perspective is still needed to capture how adolescents interpret their contexts and regulate different forms of behavior toward the goal of mitigating CC (Koskela and Paloniemi, 2023).

### 1.4 Purpose of the study

To combat CC, adolescents need to initiate, sustain, and reflect on behaviors (i.e., collective and proxy actions beyond individual ones) linked to human agency proposed by Albert Bandura (Koskela and Paloniemi, 2023). However, despite the relevance of human agency in the CC domain, the literature on this topic and its determinants is still scarce (see Cunha et al., 2025). The current data are expected to help further understand the inner workings of adolescents’ climate agency, which is relevant to setting educational practices and community interventions tailored to their needs.

This study adopted a post-positivist paradigm. Guided by Bandura’s theory of human agency (Bandura, 2018), including the triadic reciprocal causation model, it aimed to explore (i) adolescents’ climate agency modes and (ii) how personal, behavioral, and environmental determinants may facilitate or hinder the adolescents’ climate agency. As literature indicates a fluctuation in knowledge, interest, concern, or pro-environmental behavior throughout adolescence (see Kaplan and Kaplan, 2002; Lee et al., 2020; Otto et al., 2019), this study was conducted with early and middle adolescents aged between 11 and 18 (7th - 12th grade).

All considered, this study aims to expand the existing literature by answering the following research questions:

*What are the adolescents’ climate agency modes?*
*How do personal, behavioral, and environmental determinants contribute to adolescents’ climate agency?*

## 2 Materials and methods

Prior to the commencement of the study, ethical approval was obtained from the Ethics Committee for Research in Social and Human Sciences (CEICSH 112/2022) and from the Data Protection Officer of the University of Minho. All procedures were conducted in accordance with the Declaration of Helsinki. Additionally, the Portuguese Ministry of Education also provided permission to collect data in schools.

### 2.1 Participants

The research team used online (i.e., Facebook and Instagram) and face-to-face (i.e., in schools) dissemination strategies to recruit middle and high school students across various regions of the country. Participants were also recruited through snowball sampling. These methods aimed to reach a broad and diverse sample. After initial contact with the participants or their guardians, the written informed consent, including the study’s topic, general goal (i.e., explore adolescents’ actions toward environmental issues), and data collection procedure, was provided to the guardians. The CC-specific domain was not disclosed to minimize bias during data collection.

Fifty-two adolescents agreed to participate in the study that involved individual interviews (parts 1 and 2) and a focus group discussion (optional); one participant dropped out. Fifty-one (68.6% were female), aged between 11 and 18 years (Supplementary Table 1), completed the first part of the interview. Twenty-six (51.0%, 37.3% were female) were enrolled in middle school (ten participants in the 7th grade, five in the 8th grade, and eleven in the 9th grade), and 25 (49.0%, 31.4% were female) were enrolled in high school (six participants in the 10th grade, nine in the 11th grade, and ten in the 12th grade). The number of participants enrolled (i.e., 26 middle- and 25 high school students) is consistent with Warren’s (2002) recommendation that 20–30 participants are sufficient to ensure data diversity and saturation. Middle school students were aged between 11 and 16 years (*M* = 13.27; SD = 1.28), and high school students were aged between 14 and 18 years (*M* = 16.48; SD = 1.23). Participants were from nine districts of mainland Portugal and the Azores and Madeira islands, with the majority being from the northern region of the country.

Of these participants, 32 (62.7%) moved on to the second part of the interview; 15 (29.4%) were from middle school and 17 (33.3%) from high school. The sociodemographic information was similar. Middle school students were aged between 11 and 16 years (*M* = 13.73; SD = 1.33), and high school students were aged between 14 and 18 years (*M* = 16.82; SD = 0.95). The geographic diversity was maintained (i.e., seven districts of mainland Portugal and one island).

Finally, this pool of 32 adolescents was invited to participate in a focus group discussion. Of these, eight responded positively. However, due to the difficulty of coordinating the adolescents’ schedules (e.g., great variability of out-of-school activities and school schedules), only five (one female) participated in the focus group (i.e., one was attending the 10th grade, and four the 12th grade), aged between 14 and 18 years old (*M* = 16.6; SD = 1.52). These participants were from three districts of mainland Portugal.

### 2.2 Data collection

Data were collected in person or online during the second semester of the 2023/2024 school year through a semi-structured interview comprised of two parts and a focus group (see Supplementary Table 2). The interview questions were guided by Bandura’s theory of human agency (Bandura, 2018). The photo-elicitation method was used to begin the interview. This method promotes a comfortable space for discussion and participants’ involvement, facilitating a more thorough exploration of their responses (Epstein et al., 2006). The photographs presented were gathered from a single copyright-free website (Pixabay) and followed two criteria: i. depict an actual event, not too disturbing, and ii. represent the causes or consequences of CC. The participants were shown the photographs (see Supplementary Figure 1) without an explicit mention of CC. This procedure aimed to explore their knowledge of CC while avoiding the feeling that they were taking a school assessment test. After the photo-elicitation questions, every participant was asked to define CC. The interviews were interrupted for the 19 participants who were unable to define CC. The remaining 32 proceeded to the second part of the interview, which explored the climate agency modes and personal, behavioral, and environmental determinants. Interviews following the two parts of the script lasted approximately 30 min. The interviews were audio-recorded and labeled with a code for each participant and date. At the end of the interview, participants were asked to complete sociodemographic data.

The focus group was conducted after the individual interviews to complement these data by enabling participants to discuss, clarify, and build upon one another’s perspectives through group interaction. The discussion was structured following the recommendations of Krueger and Casey (2014). Two researchers (moderators) conducted the focus group through Zoom for approximately 90 min. The moderators initiated the session by explaining the rules of the focus group and developed a warm-up activity (i.e., participants were asked to choose an image that immediately came to mind when thinking or hearing about CC and share the reasons for their choice). Then, participants were encouraged to share their opinions and experiences while the moderators facilitated the discussion and kept the group focused on the central issues. Note that the moderators only asked questions of the second part of the interview script, specifically focused on their CC mitigation behaviors and determinants (see Supplementary Table 2).

### 2.3 Data analysis

Content analysis was used to analyze the individual interviews and the focus group transcriptions with the support of NVivo software. Content analysis involves identifying categories using systematic procedures (Bardin, 1996): (1) reading the interview transcript to grasp a general analysis of the data, (2) following a deductive approach to coding the data into theoretical codes and an inductive approach to identifying codes emerging from the data, and (3) processing and interpreting the data based on inferences (e.g., comparing frequencies and coded categories). As the analysis progressed, new subcategories were incorporated using participants’ own words. All transcriptions were carefully reviewed to verify whether the coded material matched the new subcategories. Participants’ responses were coded using a previously defined codebook (see Supplementary Table 3) based on Bandura’s Theory of Agency and the triadic reciprocal causation model (Bandura, 1986, 2018). Two categories were defined: climate agency modes (i.e., individual, collective, and proxy) and determinants of human functioning (i.e., personal, behavioral, and environmental). Regarding the climate agency modes, three properties (i.e., forethought, self-reactiveness, and self-reflectiveness) were defined as subcategories.

For personal determinants, three subcategories were defined according to the literature: knowledge, self-efficacy, and low outcome expectations. Specifically, for the subcategory “knowledge,” four levels were defined [i.e., no basis, beginning, developing, and proficient, based on Cleary et al. (2022)]. Moreover, other subcategories emerged from data, such as concern for the environment/planet and perceived lack of power to mitigate CC (i.e., social and political). For behavioral determinants, *a priori* subcategories were defined as CC mitigation behaviors (these were divided according to the Sectoral Solution to CC defined by the United Nations Environment Program [UNEP] - Energy Systems; Agriculture, Forestry, and Other Land Uses; Industry; Transports; and Buildings) (United Nations Environment Programme, 2024). Subcategories that emerged from the data were: collective actions, informing or influencing others about CC, lack of control, and searching for information. Finally, regarding environmental determinants, different contexts of influence, such as school, family, friends, and media, were defined *a priori*, and subcategories, such as community and lack of resources, emerged from the data (see Supplementary Table 3). The senior researcher independently reviewed the final version of the codebook to ensure its conceptual coherence and alignment with Bandura’s theoretical framework.

The coding process was conducted in three phases. First, interviews with middle school participants were coded, followed by interviews with high school participants, and lastly, the focus group. After coding the second interview with the high school students, no new categories or subcategories were identified, which suggests code saturation (Rahimi and Khatooni, 2024).

To ensure the trustworthiness of current findings (Lincoln and Guba, 1985), a second researcher independently coded around 20% of the interview transcripts. Throughout this process, coders discussed the differences, aiming to reach agreement (e.g., the subcategory “Informing or Influencing others about CC” was divided into subcategories related to specific contexts, i.e., “Family,” ‘Friends’ and “School”). The Kappa value for each (sub)category ranged between 0.86 and 1.00, which is considered “almost perfect” according to Landis and Koch (1977, p. 165). Importantly, the codebook served as a transparent and theoretically informed analytical framework. Its iterative development and review supported the researchers’ reflexive engagement with the data by making coding decisions explicit while allowing categories to be refined or added when participants’ accounts extended beyond the initial theoretical framework.

Finally, a matrix coding query crossing nodes with attributes provided the number of participants in each (sub)category by school level. The respective percentages were calculated and used to summarize data patterns. Moreover, patterns in data from individual interviews and the focus group were compared using a matrix-coding query, crossing nodes with the classified sources.

## 3 Results

This study explored adolescents’ climate agency modes and how personal, behavioral, and environmental determinants contribute to their actions. This section reports the data pattern by school level, considering the number of participants for each category and subcategory (see Supplementary Table 4). Figure 1 provides a graphical summary of findings.

**Figure 1:**
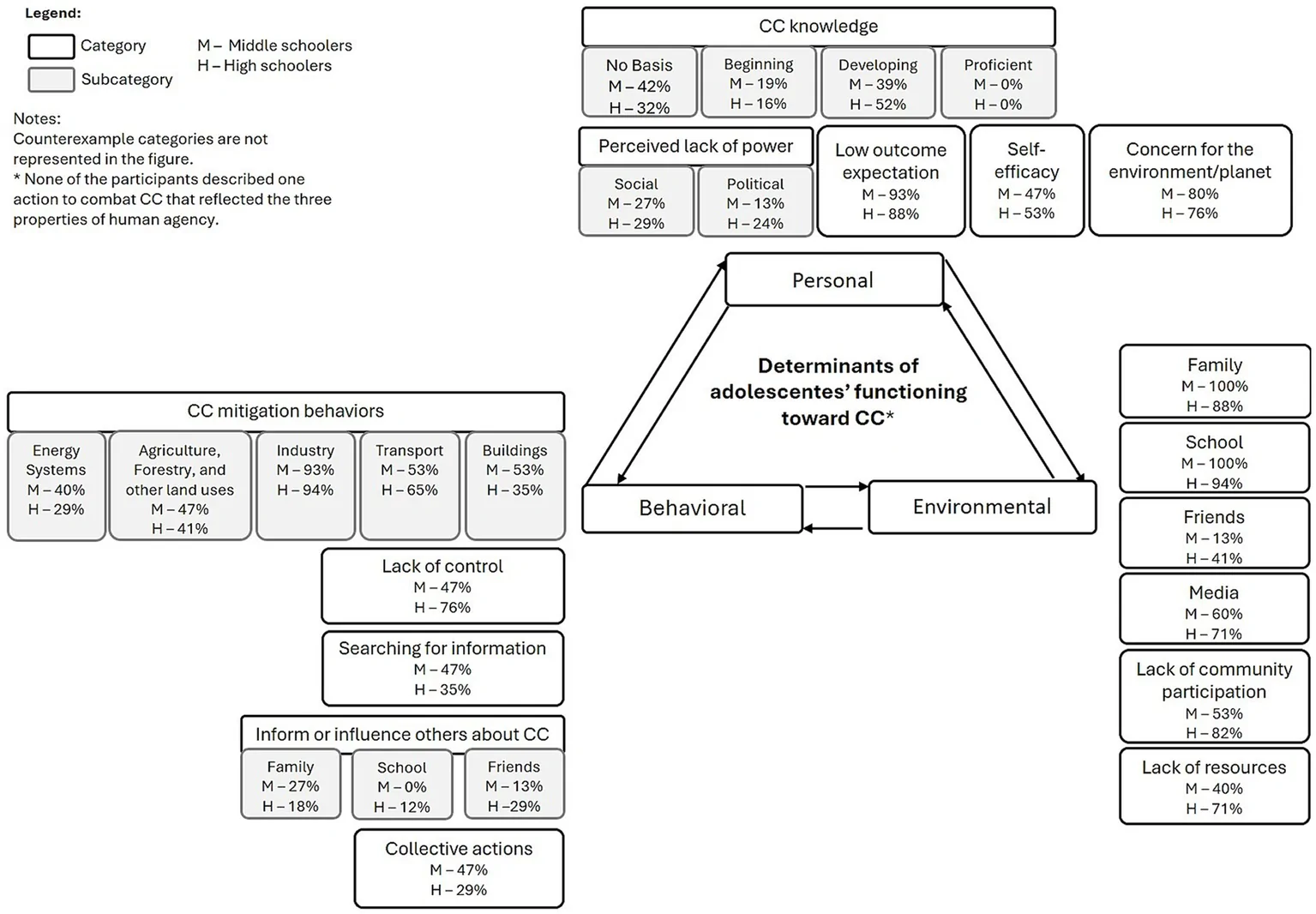
Graphical representation of findings.

### 3.1 Adolescents’ climate agency modes

During the interview, participants mentioned actions displayed in their daily lives to combat CC. They reported particular aspects associated with the properties of the individual, collective, and proxy modes of agency (i.e., forethought, self-reactiveness, and self-reflectiveness).

Concerning the forethought property of the individual agency mode, 47% of participants from middle school and 24% from high school mentioned strategies used daily to put into practice particular pro-environmental behaviors, such as reusing bags when shopping, reusing water bottles, or using a reusable bottle (e.g., keep the reusable bottle in a strategic place to avoid leaving home without it). The following utterance illustrates a participant and their family’s strategies to reuse bags when they go shopping:

“It’s the quantity [of plastic bags] that really impresses me. So, it’s something we do right here at home [reusing bags]. When we go shopping, we always keep bags in the car’s boot; this way, nobody forgets. Whenever we go to the supermarket, we just grab a bag, and it is there.” (27ER)

Participants mainly mentioned aspects associated with the self-reactiveness property (80 and 77% from middle and high school, respectively), i.e., participants reported how they put into practice some CC mitigation behaviors:

“At home, we separate our waste, put it in reusable bags, and later [when the reusable bags are full] we take it to the recycling bins instead of throwing everything into the regular trash bin.” (20ER)

Aspects associated with the self-reflectiveness property, such as reflecting on the impact of their behavior or the importance of their objectives, were shared by participants (27 and 35% from middle and high school, respectively), as the following quote illustrates:

“I think in general it’s much easier for me to decide for myself: I’m not going to throw garbage on the ground, I’m going to pick it up and put it in the right place, I’m going to try to use less electricity, I’m going to use public transportation more to save gas (…) It’s more beneficial to try to do all these little things on your own than to try to change everything and everybody. I think it’s much more unrealistic to change on a global scale than it is for us to change individually, and even though it’s the global scale that’s causing the big problem, if we change individually, we’re already making a big difference.” (22ER)

For the forethought property of the collective agency mode, percentages were higher for middle school participants (40%) than for high school participants (18%). The following utterance illustrates this participant’s understanding of actions classmates could develop to raise money to implement sustainable energy in the school:

“I also hope that schools will soon be able to adopt sustainable energy. I would like to take the initiative to have sustainable energy in the school, and I would like the students and the school to help [set initiatives]. I think we could try to suggest [a list of activities] to the students’ association (…) organize sales and projects to raise money for the school that could help install, at least, a pavilion or a room, or a group of rooms with sustainable energy, or a power station or something like that.” (26ER)

Concerning the self-reactiveness property of the collective agency mode, consistently, percentages were higher for middle school participants (47%) than for high school participants (29%). Participants reported engaging in collective actions to help the environment, stressing reasons to adopt these behaviors, such as participating in environmental-related groups (e.g., Eco-School Program, Interact Program, or environmental school clubs).

“I’m in the Eco-schools group, and we usually help collect trash. Children really think that just a piece of paper won’t do any harm, but if everyone thinks that a single piece of paper won’t do any harm, 7 billion people throwing trash on the ground would be a disaster.” (41ER)

Middle school participants did not reference aspects related to the self-reflectiveness property, and only one high school participant (6%) shared their opinion on the impact of collective actions on CC:

“I have taken part many times [actions, for example, cleaning beaches or reforestation], and I think this is one of the actions that not only have an impact in practice but also helps us to be aware of what those actions are, what the problem is, and what our behavior needs to be…” (11ER)

Regarding the proxy agency mode, one participant from each school level reported aspects associated with the forethought and self-reactiveness properties, but not the self-reflectiveness property. The teacher leader and the City Hall services were the mediators mentioned by the participants. The following utterances reflect participants’ efforts to achieve their goals through other people or entities:

Forethought: “It’s a real case. I’ve been talking to the leading teacher of my class [a teacher who manages the class issues and may have power of influence on the school principal] to help me change the situation at the school bar that sells plastic water bottles, like the kind you can only use twice. Each one of us could ask to fill our reusable bottle.” (51ER)

Self-reactiveness: “For example, we don’t have recycling bins nearby. That’s an obstacle, but I’ve also sent an e-mail to the City Hall, and they came to look at it, and there weren’t any great conditions to put them in.” (40ER)

Despite the references associated with the properties of different modes of agency, none of the participants described a CC mitigation behavior that incorporated all three properties.

### 3.2 Personal determinants of human functioning toward CC

When asked to define CC, most participants struggled to answer; 42 and 32% of middle and high school students, respectively, were unable to define the phenomenon. Of the remaining, 19 and 16% of the middle and high school answers were coded as “beginning level of knowledge”, while 39 and 52% were coded as “developing level of knowledge”. None of the participants provided a definition fitting all the criteria, and no answer achieved the proficient level.

Beginning: “I know that, for example, ah… glaciers melting, it is a CC that’s not very beneficial for us because it raises the sea level and all that.” (33ER)

Developing: “Global warming, I suppose. Global warming generates all this. Normally, people think global warming is just getting warmer, but in reality, it causes much damage, not only in terms of droughts and glaciers melting, but also floods and storms.” (14ER)

Overall, participants from both school levels recognized they needed to learn more about the topic, particularly concrete CC mitigation behaviors to implement daily. For example, “[there is the need for] more information and campaigns in schools for students so that we can understand more about the subject and more solutions to be practiced in everyday life” (12ER).

The focus group data corroborated the superficial knowledge found in the individual interviews. For example, one participant discussed the importance of reducing meat consumption at some point in the discussion. The remaining participants showed to be confused and acknowledged they were not understanding the connection to CC mitigation, as the following utterance illustrates: “I don’t know if that part about meat, I don’t know if I’m very well informed on that part, but otherwise…” (9ER). Finally, the first participant linked meat reduction to lower methane emissions from cows and clarified the point.

According to the participants’ reports, other personal determinants contributed to, or hindered, the implementation of CC mitigation behaviors. These determinants included lack of social or political power, (low) outcome expectations, or (low) self-efficacy.

Regarding the lack of social power, approximately 30% of participants from both school levels (see Figure 1) reported that people did not listen to them or that their ideas or efforts to combat CC were ignored. Overall, participants seemed hesitant, acknowledging the potential value of advocating CC mitigation to society, while simultaneously expressing a sense of limited power:

“At a societal level… hmm, yes, I could try to spread the message of less use [engage in CC mitigation behaviors]. It is a bit difficult because right now, I can’t make a big difference in influencing [people], I cannot influence many people in society, don’t you agree?” (26ER)

Regarding the lack of political power, participants (13 and 24% from middle and high school, respectively) tended to feel powerless, attributing great responsibility to governments. For example:

“Sometimes I would say a little bit of hope [is needed], because if ordinary persons cannot do the things themselves to turn everything around, and then you look… at the government not managing things well and … to the many things in society that are put aside, many issues in favor of the economy, you get a bit discouraged and start thinking: ‘if they [government] do not make efforts … what difference am I going to make [with my behaviors]?” (14ER)

This perception of a lack of power translated into a low outcome expectation, as most participants (93 and 88% from middle and high school, respectively) indicated at some point during the interview. These participants pictured the consequences of their behavior as having a minimal impact on mitigating CC. The following utterance illustrates this idea: “It is low [my impact] because it is not one person out of 8 billion or seven that’s going to make a big difference” (12ER). In the focus group, when discussing the importance of reducing meat consumption, reports indicate a similar low outcome expectation:

“Even if I decided to make it a meal that didn’t start with meat, for example, today I don’t eat (…), it wouldn’t change the fact that the animals are slaughtered anyway. In other words, it wasn’t because I didn’t eat meat today, for example, that was going to move the hundreds of thousands of animals that are dying now to other countries, to other cities. (…) It’s not just that people themselves stop eating meat, but that the people who produce it also start to decrease [the production] (…)” (12ER)

However, in the interviews, around 20% of middle school and 24% of high school students expected positive outcomes from their behavior: “Although it is on a global scale, which causes the big problem if we individually change, we are already making a big difference” (22ER).

In addition to evaluating the consequences of their behaviors, participants also judged their ability to plan and carry out CC mitigation behaviors. Despite the high percentages of participants with low expected outcomes, about 50% of participants (47 and 53% from middle and high school, respectively) reported positive judgments. For example: “I think I can help in any way. I can combat CC, (…) I don’t think there’s anything to stop me from doing it” (33ER). However, around 40% (middle school) and 41% (high school) of the participants perceived low self-efficacy, as reported by the following participant: “There are things that I cannot do, for example, because they’re things that are not up to me to solve because I do not have the skills” (2ER).

Superficial knowledge, perceived lack of power, low outcome expectations, and low self-efficacy were identified as obstacles to participants acting in the context of CC. However, most participants (80 and 76% from middle and high school, respectively) reported concern for the environment/planet and future generations. Moreover, this concern was reported as one of the main reasons for carrying out and maintaining their CC mitigation behaviors daily:

“I think it’s more of a concern, ah, that in the future, okay, we will live in, not me, but for example, my children, or the people who come after me will live in a world that is more destroyed by warming, with worse living conditions than we have in terms of nature, and so on” (8ER)

In some cases, participants mentioned this concern was prompted by watching images or news about CC (e.g., animals dying in wildfires).

### 3.3 Behavioral determinants of human functioning toward CC

Regarding their daily CC mitigation behaviors, participants mentioned behaviors related to different sectors (i.e., energy systems; agriculture, forestry and other land uses; industry; transport; and buildings). Overall, participants’ actions were related to one or up to five sectors (mainly from two or three sectors).

Specifically, participants (40 and 29% from middle and high school, respectively) mentioned saving energy by turning off lights or appliances not in use: “I am always careful. If I charged my cell phone overnight, I unplug it when I wake up in the morning. Even if I leave it on the floor, I always have to unplug it.” (5ER). Participants (47 and 41% from middle and high school, respectively) also declared they were likely to choose environmentally friendly products and/or participate in reforestation actions. One participant reported performing both behaviors, as the following utterance illustrates: “For example, (…) I choose products in the supermarket that are more respectful of the soil and ecosystems. (…) I have also often taken part in reforestation actions” (11ER).

However, the CC mitigation behaviors mentioned by most participants relate to the industry sector. Both middle (93%) and high (94%) school students mentioned recycling or reusing plastic bags, water bottles, and other materials, “for example, when we go shopping and stuff, we never buy plastic bags and stuff, we reuse them a lot” (24ER). Regarding transport, participants (53 and 65% from middle and high school, respectively) reported using public transport or walking instead of being transported in their parents’ car, especially for short distances: “I always take a public transport, I rarely take private transport, and I often choose to walk instead of taking the bus, for short distances (…)” (11ER).

Participants (53 and 35% from middle and high school, respectively) also mentioned some CC mitigation behaviors related to home management (buildings sector), such as using natural light, or reusing water in their daily lives, “For example, in the case of showers and such, when we put the water… Instead of having the water run, we put the water in a bucket, and then we can water the plants and such” (50ER). Consistently, participants in the focus group mostly mentioned behaviors associated with these categories.

Despite the CC mitigation behaviors reported by the participants, many admitted a lack of control that hindered their ability to sustain them. Looking for comfort, sloppiness or even forgetfulness when adopting CC mitigation behaviors were reported by more high schoolers (76%) than middle schoolers (47%). One typical example provided by participants during individual interviews and focus groups was taking long showers, especially in the Winter. Participants stated that despite knowing they should not take long showers, they usually take 20 to 30-min showers in Winter to warm up:

“We stop wanting to do them [CC mitigation behaviors] because maybe it doesn’t give us as much comfort… we start doing the opposite, because it gives us more comfort. In the case of bathing, in Winter, because it’s warm, we put the ecological and environmental reasons aside and maybe prioritize what’s wrong, the comfort of that moment, and maybe give up on having a better future.” (21ER)

Another typical example provided during the interviews was recycling. The participants presented recycling as one of the most acquired behaviors, but they also stated that at some point, they did not put the waste in the correct recycling bin:

“We, myself and others, try to do what we can, but often there’s a regular bin, and we end up throwing everything in there and don’t think about recycling.” (9ER)

In addition, participants (47 and 35% from middle and high school, respectively) mentioned displaying a proactive approach to researching information autonomously to pursue their interests or clarify doubts. Most of these participants reported using the internet to get information through websites, videos, and social networks. Sometimes, participants were accidentally exposed to the contents, which triggered their search as the following utterance illustrates: “(…) videos we also see on the Internet talking about how the planet is doing (…) Sometimes they come up, and I’m interested, and I research more, but I usually research when I have doubts about something” (29ER). A few participants reported asking their parents or teachers for more information. However, approximately 20% of middle and 35% of high school participants reported having no interest in learning about, researching, or discussing CC with others. For example, “I feel like I don’t do that. I don’t go looking for information on this subject because… It is not a subject I hear about that much at school and stuff” (12ER).

Furthermore, a few participants (12–29%, see Supplementary Table 4) emphasized the importance of informing, sensitizing, or influencing those around them about CC. These participants expressed a desire for social involvement, reflecting a drive to raise awareness and promote actions in various contexts of their lives, especially among family and friends.

Family: “For example, my grandparents also use plastic bags; they always reuse them, also because of our influence, because we have told them that it is good.” (24ER)

School: “my class is already trying to alert the whole school [toward the CC impacts]” (5ER)

Friends: “I try my best to inform my family, my friends, and my classmates that we have to take these measures into account, because… then, the planet is dying!” (14ER)

Finally, participants (47 and 29% from middle and high school, respectively) mentioned participating in groups to carry out collective actions to combat CC, namely planting trees, cleaning up beaches, and creating, caring for, and harvesting vegetable gardens. Participants provided examples of activities performed with their families or friends (“I do it from time to time, like with my cousins and stuff. We go and pick up the garbage we find and stuff. It is something we do a lot”, 20ER), but mainly with groups of students from their school:

“Today, for example, we had a man [at school], (…) the Trash Traveler, who traveled around the Portuguese coast and collected garbage. Then we had an activity where we collected our school’s garbage and made sculptures of it, drew pictures, and explained why.” (51ER)

### 3.4 Environmental determinants of human functioning toward CC

When asked about the context that most influenced them to combat CC, the participants mentioned their family, school, friends, and the media. However, participants revealed other determinants that negatively affected their CC mitigation behavior, such as their community or lack of resources.

At some point during the interview, most participants mentioned family and school as the primary sources of influence. Participants (100 and 88% from middle and high school, respectively) referred to the family as having a key role in their early education through initiating pro-environmental behaviors such as recycling or discussing everyday news related to the environment (“I think my parents and the school had the most influence at the first moment,” 11ER).

The school was mentioned as a continuation of their education, with participants (100 and 94% from middle and high school, respectively) reporting greater access to information through classes, lectures, group work on the subject, or discussions with teachers.

“Maybe the school, because the teachers and so on talk about it often, and sometimes we have lectures too. (…) And I think that helps us understand the problems we have [with CC] and how we can avoid them.” (24ER)

Moreover, part of these participants (47 and 29% from middle and high school, respectively) also highlighted the positive role of the environmental initiatives (e.g., creating and caring for vegetable gardens, cleaning up beaches or the streets near the school) organized by their school (e.g., through the Eco-School Program or the Environment Club) to help them be more aware of environmental problems such as CC.

However, participants (33 and 47% from middle and high school, respectively) also reported a negative influence of school when it lacks concrete measures inside the school or effective teaching methods. One participant explained this point of view:

“At school, we have several talks about this, but they [the school] do nothing specific. They do not apply any measures inside [the school] for us to do anything about it. (…) I think that if they really pushed us at school on this subject, and the teachers and so on, they’d be able to explain other ways for us to apply at home and in our daily lives.” (19ER).

Participants also mentioned friends as another influential determinant. Around 13% of middle and 41% of high school students reported that their friends positively influenced their behaviors or thoughts about CC:

“I’d say that maybe it’s more my friends who exert this influence [to display more CC mitigation behaviors] (…). I think it also varies according to age, it varies a lot from person to person, and it varies according to the education we’ve had; I think that first my parents and school had more influence, but now maybe the people I have relationships with, my age, my friends, have more influence.” (11ER)

However, a few participants mentioned that this influence can sometimes be harmful. One participant shared that sometimes individuals who act to protect the environment and slow CC (e.g., by putting garbage in the proper recycling bin) are made fun of by their peers, which is embarrassing. This idea was addressed and further corroborated throughout the focus group:

“In my old group of friends, most people threw garbage on the ground, I believe, because of each other’s influence. (…) I think friends influence our behavior a lot, because we spend much of our time with them.” (12ER)

Participants (60 and 71% from middle and high school, respectively) reported how media (e.g., television and social media posts) positively influenced their thoughts, behaviors, or feelings to act to slow down CC. One participant revealed that knowledge acquired through the internet and social networks allowed them to look for better ways of acting toward CC, that is:

“I think that social media and the Internet also influence a lot (…) because, for example, if they [environmental influencers] talk about a certain company that does not waste energy and uses solar energy, I look for that company because I know that it does not waste the environment so much” (26ER).

Conversely, a few high school participants (12%) referred that the media could have a negative influence (see Supplementary Table 4). They illustrated this idea by citing famous influencers who advocate for consumption (e.g., clothing, technology).

“I think it’s a lot about the media, about TikTok. Sometimes you go to TikTok, and you see those girls doing the hauls, like “oh, today I have, I bought something at Zara [clothing store]”, and I think, oh, I want it too, I’m going to buy it at Zara. In my opinion, I also think we are very influenced [negatively].” (4ER)

Moreover, participants (53 and 82% from middle and high school, respectively) mentioned the relevance of the surrounding community. They explained the need for more people from their community to be involved in the combat against CC to feel motivated to adopt more collective actions. For example, “And so it is also a bit about having more people to do things when it comes to not so much personal actions, but in the case of cleaning beaches, planting trees, those two are the main ones” (22ER).

Finally, participants (40 and 71% from middle and high school, respectively) mentioned a lack of economic (e.g., high prices of organic products or solar panels) and material (e.g., access to recycling bins, public transportation) resources as obstacles to combating CC. The following utterance illustrates the scarcity of public transport:

“I think people could make an effort to, it does not have to be all the time, but at least sometimes (…) try to use public transport (…). Unfortunately, I do not do it anymore because, in the neighborhood where I live, there is almost no [public] transport; I also think the [city council] could provide more [public] transport. Cities could focus more on that.” (5ER)

However, a small percentage of participants from both school levels (13 and 18% from middle and high school, respectively) reported positive developments in accessing resources (e.g., recycling bins) and considered access to resources as a facilitator. This idea was corroborated in the focus group discussion.

“Nowadays, we already have recycling bags at school. Schools are already talking about reusable bottles; supermarkets are selling them more easily; I think it is really the people saying I want to do this; I want to be able to (…) help nature and not pollute.” (4ER)

“As for recycling, it’s something we’ve always done here at home and will continue to do. Besides, the town hall gives people individual bins, so it’s much easier to recycle”. (21ER)

## 4 Discussion

Extant literature has highlighted the need for adolescents to develop an agent role toward CC (Sanson et al., 2019; Baldwin et al., 2023). In this vein, through the lens of Bandura’s social cognitive theory (Bandura, 2006, 2018), the current study explored adolescents’ climate agency modes, focusing on expressions of individual, proxy, and collective actions toward CC mitigation. Additionally, the study mapped their personal, behavioral, and environmental determinants.

This theoretical framework was a helpful tool for further understanding the adolescents’ CC mitigation behavior. Current data indicate that adolescents mainly engage in individual actions. Considering that individual actions alone are insufficient to address global issues such as CC (Bandura and Cherry, 2020; Koskela and Paloniemi, 2023), it is noteworthy that there was limited evidence that participants expanded their influence through collective or proxy actions. Moreover, no CC mitigation behavior described by participants simultaneously met all three properties (see Supplementary Table 4). It is promising that participants mentioned carrying out various CC mitigation behaviors (indicating some processes of the self-reactiveness property). However, they do not seem to set action plans and reflect on their CC mitigation behaviors or their effectiveness, suggesting poor forethought and self-reflectiveness across individual, collective, and proxy climate agency modes. These findings warrant attention as, according to Bandura (2018), “forethought enables people to transcend the dictates of their immediate environment and to shape and regulate the present to realize desired futures” (p. 131), and self-reflectiveness allows “individuals [to] address conflicts between alternative courses of action and competing values and favor one course over another”. In addition, a high percentage of adolescents reported sloppiness or forgetfulness as reasons for not sustaining their CC mitigation behaviors, also reflecting a poor self-reactiveness property. The data analysis using the three properties of the human agency construct suggests that participants’ CC mitigation behaviors were not self-regulatory driven, indicating that these participants’ climate agency is not developing in a multidimensional way according to Bandura’s framework. As prior authors have noted, self-regulatory processes and strategies (reflected in the properties of human agency in this study) can be key to explaining and overcoming the gaps between individuals’ knowledge or intentions and the adoption of CC mitigation behaviors (Colombo et al., 2023a,b). A thorough analysis of the personal, behavioral, and environmental determinants may deepen the understanding of the current findings.

### 4.1 Personal determinants of human functioning toward CC

Findings revealed personal determinants of participants’ CC mitigation behavior as follows: knowledge, lack of power, (low) outcome expectations, (low) self-efficacy, and environmental concern. Results showed that many adolescents (32–42%) were unable to define CC, whereas the remaining defined the concept superficially. Besides, participants generally expressed a need to learn more about the topic. These findings are somewhat consistent with prior studies (e.g., Chang and Pascua, 2016; Doğru and Yüzbaşıoğlu, 2023; Karpudewan et al., 2015; Özcan Tan and Demirkaya, 2025), including the UNICEF-Gallup survey (UNICEF, 2023). Conducted in 55 countries and enrolling participants aged 15 to 24, this survey found that around half of young people cannot define CC, despite having heard about it. Together, these data are particularly worrying because, according to UNESCO (2022), CC education in Portugal is embedded in the school curriculum. Education begins by discussing concepts like air quality and pollution for younger students and progresses in middle school to subjects such as Geography, Natural sciences, and Physical chemistry, addressing human impacts on the climate and natural hazards (note that in the subject of Geography at 9th grade, CC has greater prominence than in prior grades). In high school, topics like global warming and the greenhouse effect are explored in Geography, Chemistry, Physics, Geology, and Biology (UNESCO, 2022). However, despite efforts to embed CC content in the curriculum, these are not yielding fruitful results. Current findings merit the attention of educators and educational authorities as CC knowledge (when combined with other components) is one of the key elements likely to contribute to behavior change (see Hohenhaus et al., 2023). As Rousell and Cutter-Mackenzie-Knowles (2020) alert, education needs to adopt a more comprehensive and practical approach to improve knowledge of diverse CC mitigation behaviors.

In addition, participants mentioned a lack of social and political power to implement systemic changes in CC. As recently reported by Rebelo et al. (2025), young people feel their individual actions are insufficient to bring about change in society and perceive a lack of power to overcome structural barriers (e.g., improving public transport networks). This lack of social and political power may be related to young people’s perception that governments are primarily responsible for combating CC (Corner et al., 2015) and to feelings of frustration stemming from unequal power (Rebelo et al., 2025; Hickman et al., 2021). Relatedly, in a study conducted with youth activists from an environmental civil disobedience group (Stuart, 2022), participants strongly believed that individual actions alone are not sufficient and that only governmental intervention can deliver the systemic changes needed to address CC. Despite their low expected outcomes for individual action, the activists’ pro-environmental values and identity supported their motivation to stay committed to a low-carbon lifestyle. Similarly, in the present study, most adolescents perceived low outcomes from their own CC mitigation behaviors. However, their reasoning was that everyone needs to adopt pro-environmental behaviors; otherwise, their CC mitigation actions are useless. These findings also align with previous studies, indicating that young people who feel limited capacity to make a difference are likely to become less involved in this issue (Corner et al., 2015; Muller and Wood, 2021). In Brown and Krettenauer’s (2025) study, this is one of the main reasons for disengaged adolescents to justify their inaction. According to Shell (2023), theoretically, for adolescents to feel motivated and take action toward CC, they must visualize the contingent relationships of outcome expectations: realize that behaviors lead to results they value, while increasing their self-efficacy. Participants’ difficulties in perceiving significant outcomes of their CC mitigation behaviors may help explain that only around half adolescents reported CC self-efficacy (see Supplementary Table 4). These findings are not surprising considering that adolescents may understand their individual efforts as a “drop in the ocean” (Lorenzoni et al., 2007). Altogether, current and prior data indicate the importance of promoting young people’s involvement in community actions and their willingness to generate social and political influence in the face of CC (Rebelo et al., 2025). In a global environmental problem such as CC, collective efficacy is particularly relevant (Bandura and Cherry, 2020; Ojala, 2023); however, this determinant was not found in the participants’ discourses. Current data underscore the importance of providing adolescents with both individual and collective experiences to combat CC (Buttigieg and Pace, 2013), helping them understand the effects of each CC mitigation behavior and visualize the positive outcomes, while reinforcing their CC efficacy (Shell, 2023).

Finally, more than 75% of the participants expressed concern for the environment, the planet, and the future of the world’s population; moreover, some mentioned images or news visualization as the trigger of this concern. As Galway et al. (2021) stated, young people’s concerns are currently mainly fueled by the images they see in the media and by the thoughts and conversations about the future of the planet. Prior literature (Galway et al., 2021; Sanson et al., 2019; Stevenson et al., 2018) found that CC concern is positively related to greater involvement in pro-environmental behaviors; however, even though concern may lead to action, it is important to note that, depending on the adolescents’ characteristics (e.g., low self-efficacy or defensive worldviews), shocking images or news can have a detrimental impact on youth CC mitigation behavior (see Félix et al., 2024). Hence, educators could consider identifying and discussing with students the origins of their concerns about CC to promote rather than undermine effective CC mitigation behaviors.

### 4.2 Behavioral determinants of human functioning toward CC

Current findings revealed that participants reported engaging in various CC mitigation behaviors daily. All sectors of the solution proposed by the United Nations Environment Programme (2024) to mitigate CC were identified. However, behaviors related to the sectors of Industry, Transport, and Buildings were the most frequently identified in participants’ discourses. Consistent with prior studies (Brown and Krettenauer, 2025; Otto et al., 2015), participants reported behaviors particularly related to recycling (Industry sector) and public transport (Transport sector). Notwithstanding, there is room to encourage adolescents to extend their behaviors to other sectors of the CC mitigation solution as outlined by the United Nations Environment Programme (2024). For example, opening the window to use natural light or reducing meat consumption are behaviors within the reach of adolescents that just a few have mentioned. Considering these data, the Sectoral Solution framework (United Nations Environment Programme, 2024) can be a valuable tool for educators to help students plan, maintain, and reflect on CC mitigation behaviors. Moreover, it would be useful to help students identify low, moderate, and high-impact CC mitigation behaviors (Ivanova et al., 2020; Wynes and Nicholas, 2017), as they tend to overestimate the impact of recycling, which they usually do more often, while underestimating the impact of reducing meat consumption on mitigating CC (Baldwin et al., 2023; Pickering et al., 2020, 2021).

Despite the positive CC mitigation behaviors reported, participants mentioned other factors, such as carelessness, sloppiness, forgetfulness, or seeking immediate comfort (e.g., taking long hot showers) that hinder their CC mitigation efforts. This lack of control can be associated with unintentional or automatic behaviors (Russell et al., 2017), affecting young people’s self-regulation regarding CC mitigation behaviors. Ultimately, these dysfunctional processes may limit the adolescents’ climate agency (Bandura, 2018; Koskela and Paloniemi, 2023). This finding stresses the relevance of creating opportunities for continuous learning and intentional reflection (Koskela and Paloniemi, 2023). Educators could consider delivering intentional training to adolescents, conveying the overall message that, beyond planning, self-monitoring of CC mitigation behavior is also necessary to become agents.

On the other hand, participants mentioned searching for information on CC when they have doubts or are curious about the topic. Although this is a positive behavior, it is important to note that most participants reported searching for information through websites, videos, and social networks without taking special care to use reliable sources. Adolescents may need help to develop their critical thinking skills to properly evaluate and select reliable information (Cooper, 2011). This is particularly important in the domain of CC, in which media often provide misleading information (Dawson and Carson, 2013; Thomas et al., 2022). As prior literature reports (Du et al., 2023; Thacker, 2024), when exposed to new information, young people must perceive it as interesting and valuable to be motivated to learn more and research about it. However, current findings also revealed that some adolescents reported no interest in the CC topic. This lack of interest is linked to lower pro-environmental behaviors (Brown and Krettenauer, 2025) and requires further research to understand the reasons behind this disengagement from CC, with tangible impacts on humanity in the near future.

Just a few participants reported efforts to inform and influence others about CC, including family, school, and friends. The limited number of participants reporting these actions is consistent with their superficial CC knowledge. As Žukauskienė et al. (2021) report, young people’s knowledge of how to act on CC is important for expressing their own intentions and actions, as well as for influencing their parents to engage in pro-environmental behaviors (see also Lawson et al., 2019).

Lastly, participants also mentioned engaging in collective actions, primarily associated with activities developed within the school context. Therefore, their collective participation seems to result from the schools’ planning of environmental activities, rather than from the adolescents’ intention to work together toward a CC mitigation goal (see Bandura, 2018). While the collective climate agency is lacking, as Wu and Otsuka (2021) found, out-of-class activities in schools (e.g., enrollment in clubs, activities, and environmental projects) are nevertheless likely to encourage adolescents to engage in pro-environmental behaviors. These activities enable adolescents to manage their negative emotions and concerns associated with CC (Ojala, 2023) and foster their willingness to engage in prosocial behaviors (Du et al., 2023). Educators and school directors could consider creating opportunities to reinforce the CC mitigation behaviors adolescents adopt collectively and help them connect these actions to their impact on CC (Toivonen, 2022). As literature suggests (e.g., Pickering et al., 2020), these intentional efforts help adolescents integrate collective actions into their lifestyles from an early age.

### 4.3 Environmental determinants of human functioning toward CC

Current data reveal that contexts (family, school, peer groups, and media) surrounding adolescents play crucial roles in their CC mitigation behavior. The family and school were reported as the leading positive influencers. Consistent with previous studies (Collado et al., 2019; Žukauskienė et al., 2021), family was identified as crucial for introducing pro-environmental behaviors, such as recycling and discussing CC-related daily news. Interestingly, prior research has shown that family discussions about CC are more strongly associated with their children’s climate behaviors than parents’ own climate behaviors (Lawson et al., 2019). Here, mass media (e.g., TV news) may facilitate family conversations (Wilson, 2004) on topics some parents struggle with (e.g., CC as a source of anxiety; Baker et al., 2021).

Moreover, schools complement the family’s CC education by providing scientific information through lectures, group work, and teacher discussions as reported in prior studies (Strickhouser et al., 2017; Varela et al., 2020). Notwithstanding, current participants criticized the schoolwork in this regard, suggesting that a more practical and engaging approach to CC could enhance the effectiveness of their behaviors. According to Valdez et al. (2018), young people are likely to discuss CC with teachers, but contrary to the data on family discussions (Lawson et al., 2019), this educational activity does not translate into adolescents developing CC mitigation behaviors. These findings underscore the importance of schools integrating essential (e.g., knowledge, self-efficacy) and complementary (e.g., goal setting, peer interactions, and nature connectedness) elements of CC interventions to promote adolescent behavioral changes (Hohenhaus et al., 2023; see also Monroe et al., 2017). Current findings also reveal the need to ground CC teaching practices and school environmental projects on solid theoretical frameworks. Bandura’s agency theory in particular, would help adolescents controlling and continuously improving their CC mitigation behaviors while broadening their agency to collective and proxy modes.

Friends were mentioned by middle schoolers and high schoolers as having, mainly, a positive influence, while a few participants provided worrying examples of a negative influence. Positive peer influences are consistent with previous studies reporting that friends or peers play an important role in young people’s interest and involvement with CC (Corner et al., 2015; Valdez et al., 2018). For example, Long et al. (2014) argue that friends can promote positive changes in their counterparts’ behaviors regarding waste and recycling. However, current findings, especially those from the focus group, stressed a negative influence (e.g., throwing garbage away). Schools and families could consider implementing strategies that encourage friends to discuss CC mitigation behaviors and help them cope with peer pressure.

Many participants identified the media as a positive influence on their CC mitigation behavior. Participants reported using the media (internet, news, and social networks) to learn more about CC and how to act. These findings corroborate prior research reporting positive relationships between media exposure and college students’ pro-environmental behaviors (e.g., Li and Noor, 2022). However, a few high schoolers pointed out that social media, particularly videos from famous influencers, has a negative influence because it promotes consumerism. Both data are consistent with the literature alerting to the uncertain roles CC media can play, such as entertainment, education, activism, or misinformation (Denault et al., 2024; Hautea et al., 2021). In this context, it is important that teachers and family members discuss with adolescents the need to critically evaluate information (Valdez et al., 2018; Wu and Otsuka, 2021).

Lack of community participation and lack of resources were also perceived as negative influences on CC mitigation behaviors. Participants expressed that a lack of community involvement discourages their collective behavior. This finding suggests that community-level social norms, beyond family and friends (e.g., Busch et al., 2019; Collado et al., 2019), can undermine adolescents’ motivation to engage in CC mitigation behaviors. Beyond individual efforts, the participation of their community in collective actions would help foster collective efficacy, which is highly relevant to sustaining efforts toward a global challenge such as CC (Jugert et al., 2016). Additionally, the lack of economic and/or material resources was identified as a significant obstacle, highlighting the need for better infrastructure and resources to support pro-environmental behaviors. This finding aligns with a study by Thomas et al. (2022), which argues that even for adolescents with the autonomy to make lifestyle decisions, structural barriers, such as a lack of public transportation, make it challenging to adopt pro-environmental behaviors. Interestingly, Brown and Krettenauer (2025) found that adolescent “disengagers” often cite structural barriers as excuses for inaction, whereas “engagers” recognize them as challenges and remain committed to seeking alternative solutions. These data support the need to follow divergent approaches to achieve CC mitigation goals when training adolescents (e.g., practicing proxy climate agency strategies such as writing an email to City Hall to request improvements in transport networks, summarizing the challenges, and providing suggestions; Koskela and Paloniemi, 2023). For instance, in Portugal, some schools have joined participatory budgeting initiatives that require a high degree of agency from adolescents, as they must work together from identifying needs in their school or municipality to advocating for their project proposal (Cunha et al., 2026). Adopting a flexible approach to CC mitigation is key to weakening the negative impact of environmental barriers and fostering adolescents’ self-efficacy, a strong predictor of CC mitigation behavior (Corner et al., 2015; Baldwin et al., 2023).

### 4.4 Middle versus high school results

The matrix coding query crossing each (sub)category by school levels revealed some differences in data patterns. Salient differences for climate agency modes and properties, as well as for each determinant (i.e., personal, behavioral, and environmental) are discussed hereafter.

It is noteworthy that, despite their more advanced developmental stage and growing capacity for abstract thinking during adolescence, high schoolers’ reports did not indicate a more developed climate agency than those of middle school students. Specifically, at a stage when they are starting to connect more broadly with society and gain independence (Ojala, 2023), they surprisingly showed lower collective climate agency. Additionally, high schoolers reported fewer self-regulatory skills related to the forethought and self-reflectiveness properties (of individual and collective modes) than their counterparts. When considered alongside the remaining findings discussed below, these differences suggest that contextual factors (e.g., academic demands and school-led environmental initiatives) may play a more prominent role than personal factors (e.g., cognitive maturation) in explaining variations in climate agency and their properties.

Concerning personal determinants, a higher percentage of high (52%) than of middle (39%) school students reached a developing knowledge level, and a lower percentage of high (32%) than middle (42%) school students could not define CC. These differences may be associated with the gradual integration of the topic into the school curriculum over the years (UNESCO, 2022). Despite this improvement, adolescents’ poor knowledge in high school remains a relevant issue that educators must address. These findings suggest the need to develop effective strategies to deliver CC content throughout the school curriculum.

Focusing on behavioral determinants, data indicate a lower percentage of behaviors related to “energy systems” and “building” subcategories among high school participants than among their counterparts. Most of the behaviors mentioned in the “buildings” subcategory are related to water conservation. For example, in the focus group, high schoolers reported taking long showers, especially during Winter (which also requires extra energy to heat the water). Consistently, these participants reported a higher unwillingness to engage in CC mitigation behaviors than their counterparts, citing laziness or forgetfulness as reasons. These findings suggest that older participants may lack control over their actions, which may be related to a search for comfort (e.g., taking long hot showers) or a lack of interest (e.g., forgetfulness of pro-environmental behaviors). These data reinforce the need to help adolescents’ efforts to monitor and sustain CC mitigation behaviors (Koskela and Paloniemi, 2023), particularly among older ones.

Another notable difference regarding the behavioral determinants is the search for information. High school students (35%) are less likely than middle school students (47%) to search for information through the media or by talking to others when they are curious or have doubts about CC. This seems somewhat consistent with previous data on the category of lack of control. More high school (76%) than middle school (47%) students put less effort into sustaining their CC mitigation behaviors. Moreover, middle school students (47%), compared with their counterparts (29%), reported being more involved in collective actions, such as group environmental activities, particularly those that take place within the school context. Together, these findings may be explained by two factors. The development of an interest in social and global issues among younger participants, likely to occur at this age (Holden, 2007), and a lack of interest among older participants, associated with a decreased willingness to act (Lee et al., 2020). For example, Kaplan and Kaplan (2002) characterize this decrease in older adolescents’ willingness to act as a period of “time out” regarding the development of environmental concerns. Other researchers termed “adolescent dip” the decreased interest and involvement in environmental issues in the adolescent phase (Kaplan and Kaplan, 2002; Otto et al., 2019). This decrease in interest may be related to other priorities in the lives of older adolescents, such as focusing on academic tasks and exams (Brown and Krettenauer, 2025; Cunha et al., 2026). At the same time, as Cunha et al. (2026) found, the number of environmental initiatives organized by Portuguese schools for high school students is smaller than that for middle school students, which may hinder their participation in environmental issues, such as CC, specifically collective actions.

Interestingly, more high schoolers (29 and 12%) than their younger counterparts (13 and 0%) reported efforts to inform or influence their school peers or staff as a way to act on CC (see Supplementary Table 4). The percentages at both school levels are low; however, the contrast in the data pattern may reflect the need expressed by high school students to involve more people around them in the combat against CC, as discussed below.

Concerning environmental determinants, salient differences were identified in the following categories: friends’ influence, lack of community participation, and lack of resources. The high school students’ reports in all these categories were higher than those of their counterparts. Regarding the category “friends,” the finding is surprising, as Koessler et al. (2022) found that younger adolescents (8th grade) were more susceptible to changing their climate-friendly behavior (i.e., donating to set up a CO2 offsetting scheme) than older adolescents. However, note that the types of behaviors reported in the current study differ from those used by Koessler et al. (2022) to measure climate-friendly behavior. Negative influence of friends was also mentioned by more high schoolers than middle schoolers, which is even more surprising, as prior literature shows that adolescents between 14 and 18 years old are more resistant to peer pressure concerning negative behaviors than those between 10 and 14 years old (e.g., Steinberg and Monahan, 2007).

Interestingly, more high school than middle school students reported a lack of community participation. The former highlighted, more than their counterparts, the need for more people around them to be involved in the combat against CC through collective actions (e.g., cleaning up beaches and planting trees). This finding is somewhat consistent with the set of evidence on civic engagement (e.g., pro-environmental and prosocial behaviors) summarized by Wray-Lake and Ballard (2023). These authors argue that declines in civic engagement among adolescents may be related to declines in contextual variables such as family support or neighborhood climate (e.g., “Most people in my neighborhood try to make this a good place to live”; Wray-Lake and Sloper, 2016). Taken together, these findings may prompt educators to reflect on how to promote adolescents’ collective actions within their communities. This strategy could help sustain this behavior over time.

Finally, reports on a lack of economic (e.g., high prices of organic products) and/or material (e.g., public transportation) resources were more pronounced among older participants, who mentioned this limitation as an obstacle to acting more effectively toward CC. As prior research has shown, older adolescents are more likely to make food choices, engage in food acquisition (Ziegler et al., 2021), and use public transport more frequently and over longer distances as they gain greater autonomy (e.g., Jafari et al., 2025). This may help explain why a higher percentage of high schoolers have more awareness of the related barriers than middle schoolers. This developmental stage presents an opportunity to challenge adolescents to reflect on changes in their lifestyles as they gain more autonomy and to readjust their action plans to combat CC, in line with the properties of human agency (see Bandura, 2018; Koskela and Paloniemi, 2023).

### 4.5 Strengths, limitations, and future research

The current study provides a broad understanding of adolescents’ CC mitigation behaviors by applying the theoretical lens of human agency (Bandura, 2018). Adopting this framework showed that, although individual CC mitigation behaviors coexist with some forms of collective action, adolescents’ reports suggest limited intentionality, involvement in decision-making, or long-term engagement with this global issue. The specific gaps in the three properties of each agency mode merit educators’ attention. This study also provided a comprehensive picture of the complex net of relationships among personal, behavioral, and environmental determinants and the adolescents’ functioning toward CC. This knowledge provides implications for researchers and practitioners across educational, social, and policy settings. Overall, findings stress the need to work with adolescents on various self-regulatory processes associated with each human agency property across different contexts (e.g., using intentional strategies to reach their goals and monitor their progress). The school was recognized as a privileged context for promoting adolescents’ climate agency modes. However, educators and school administrators may consider better articulating CC teaching throughout the educational process and implementing environmental projects that actively involve families and the community.

In addition to these theoretical contributions and valuable insights for educational practice, this study also has methodological strengths. First, two data collection methods were used. Individual interviews were conducted with all participants, followed by a focus group, which corroborated the interview data and enhanced the trustworthiness of the findings. Moreover, the use of the photo-elicitation method facilitated participants’ involvement (Epstein et al., 2006) and allowed exploring CC knowledge without the bias of instruments that assess recall or recognition of information (e.g., items as “CO2 causes CC”; see Busch et al., 2019). Finally, code saturation was achieved, suggesting that the collected data captured the complexity of the phenomenon (Rahimi and Khatooni, 2024).

Despite the mentioned strengths, it is important to recognize some limitations of the current research. First, findings cannot be generalized, as they reflect the specific perceptions and experiences of the participating adolescents. Moreover, while the Intergovernmental Panel on Climate Change definition used for developing CC knowledge levels aligns with the Portuguese curriculum, it may not be appropriate in countries where CC is addressed differently. Second, despite achieving code saturation, we cannot guarantee meaning saturation (Rahimi and Khatooni, 2024). Third, sex representation was not balanced. In addition, only one focus group was conducted with high school participants. Future research could include participants from additional regions of the country (e.g., regions most affected by CC) and balance sex to understand whether the data pattern is maintained.

Finally, future research could incorporate teachers’ and families’ perspectives on their role in promoting climate agency to triangulate with adolescents’ data. These sources of information could help further understand the complexity of the reciprocal relationships among personal, behavioral, and environmental determinants of adolescents’ climate agency. Moreover, future research could explore in greater depth some overlooked determinants, such as the influence of friends at different stages of adolescence, the role of media (e.g., exposure to CC information or disinformation on social media and family discussions about CC-related news), and the surrounding community that participants mentioned. Notwithstanding, current findings provide a solid ground for testing a first model of personal, behavioral, and environmental predictors of adolescents’ climate agency.

## Data Availability

The datasets presented in this article are not readily available due to privacy or ethical restrictions. Requests to access the datasets should be directed to jcunha@psi.uminho.pt.
