# Supplementary material for: Unveiling adolescents’ climate agency modes and their personal, behavioral, and environmental determinants: a qualitative study
Source: Front Psychol. 2026 Sep 17;17:1882370. doi: 10.3389/fpsyg.2026.1882370 (PMC13628779; doi:10.3389/fpsyg.2026.1882370)
Supplement: Supplementary file 1 [file Supplementary_file_1.docx]

Supplementary Material

Unveiling adolescents’ climate agency modes and their personal, behavioral, and environmental determinants: a qualitative study

Jennifer Cunha^1*^, Rafaela Carneiro^1^, Marcelo Félix^1^, Pedro Rosário^1^

^1^Psychology Research Center, School of Psychology, University of Minho, Braga, Portugal

*** Correspondence:**
Corresponding Author
jcunha@psi.uminho.pt

Supplementary Materials (Tables and Figure) are provided in the following pages.

# 1 Supplementary Tables

## 1.1 Supplementary Table 1. Participants’ demographic information.

| Code | Gender | Grade | Age (years) | Region of the Country | Area Classification* |
| --- | --- | --- | --- | --- | --- |
| 1ER | Female | 10th | 15 | District A | Predominantly Rural |
| 2ER | Female | 9th | 14 | District B | Predominantly Rural |
| 3ER | Male | 10th | 15 | District A | Predominantly Rural |
| 4ER | Female | 11th | 16 | District C | Predominantly Urban |
| 5ER | Female | 11th | 16 | District C | Predominantly Urban |
| 6ER | Male | 11th | 16 | District A | Predominantly Rural |
| 7ER | Female | 8th | 13 | District A | Predominantly Rural |
| 8ER | Female | 10th | 15 | District A | Predominantly Rural |
| 9ER | Male | 11th | 16 | District C | Predominantly Urban |
| 10ER | Female | 8th | 13 | District C | Predominantly Urban |
| 11ER | Female | 12th | 17 | District D | Predominantly Urban |
| 12ER | Male | 9th | 14 | District E | Moderately Urban |
| 13ER | Female | 7th | 12 | District C | Predominantly Urban |
| 14ER | Female | 12th | 18 | District E | Moderately Urban |
| 15ER | Male | 7th | 11 | District C | Predominantly Urban |
| 16ER | Female | 8th | 13 | District A | Predominantly Rural |
| 17ER | Male | 9th | 14 | District F | Moderately Urban |
| 18ER | Male | 7th | 12 | District C | Predominantly Urban |
| 19ER | Female | 9th | 14 | District C | Predominantly Urban |
| 20ER | Female | 9th | 15 | District A | Predominantly Rural |
| 21ER | Male | 11th | 16 | District C | Predominantly Urban |
| 22ER | Male | 11th | 16 | District C | Predominantly Urban |
| 23ER | Male | 7th | 12 | District C | Predominantly Urban |
| 24ER | Female | 9th | 16 | District C | Predominantly Urban |
| 25ER | Male | 10th | 14 | District E | Moderately Urban |
| 26ER | Female | 7th | 13 | District C | Predominantly Urban |
| 27ER | Female | 12th | 17 | District C | Predominantly Urban |
| 28ER | Female | 12th | 18 | District G | Moderately Urban |
| 29ER | Female | 7th | 12 | District C | Predominantly Urban |
| 30ER | Female | 10th | 15 | District C | Predominantly Urban |
| 31ER | Female | 9th | 14 | District C | Predominantly Urban |
| 32ER | Female | 9th | 14 | District C | Predominantly Urban |
| 33ER | Male | 11th | 17 | District C | Predominantly Urban |
| 34ER | Female | 9th | 15 | District C | Predominantly Urban |
| 35ER | Male | 12th | 17 | District E | Moderately Urban |
| 36ER | Female | 12th | 18 | District C | Predominantly Urban |
| 37ER | Female | 12th | 18 | District E | Moderately Urban |
| 38ER | Male | 8th | 14 | District H | Predominantly Rural |
| 39ER | Female | 12th | 18 | District I | Moderately Urban |
| 40ER | Female | 12th | 18 | District C | Predominantly Urban |
| 41ER | Female | 7th | 13 | District C | Predominantly Urban |
| 42ER | Male | 10th | 15 | District A | Predominantly Rural |
| 43ER | Female | 7th | 12 | District B | Predominantly Rural |
| 44ER | Female | 8th | 13 | District H | Predominantly Rural |
| 45ER | Female | 11th | 17 | District J | Moderately Urban |
| 46ER | Female | 7th | 11 | District K | Predominantly Rural |
| 47ER | Female | 12th | 18 | District K | Predominantly Rural |
| 48ER | Female | 11th | 16 | District K | Predominantly Rural |
| 49ER | Female | 7th | 12 | District D | Predominantly Urban |
| 50ER | Male | 9th | 15 | District C | Predominantly Urban |
| 51ER | Female | 9th | 14 | District D | Predominantly Urban |

*Note*. Letters were assigned to each district to prevent participants' identification. *Following the Portuguese Statistics (Instituto Nacional de Estatística, n.d.) categorization of urban areas, the participants' schools were located in predominantly urban (*n* = 27), moderately urban (*n* = 9), and predominantly rural (*n* = 15) areas.

## 1.2 Supplementary Table 2. Interview script.

| Interview part | Type of questions | Questions |
| --- | --- | --- |
| Part 1 | Photo-elicitation questions on CC | 1. What do you see in these pictures? |
|  |  | 2. What do you think this set of images might represent? |
|  | Questions about CC knowledge | 1. What is climate change?^*^ |
|  |  | 2. What do you think is at the root of climate change? |
| Part 2 | Questions to explore the adolescents’ climate agency modes and determinants | 1. What behaviors or measures can be taken to combat climate change?^*^ |
|  |  | 2. Which of these behaviors have you done or are you currently doing? (If so, ask for examples from the last week.) For example, in the last week, what behaviors have you done? What, if anything, did you do or think about that helped you behave that way?^*^ |
|  |  | 3. What about the people around you? (Explore family, friends, school, and community). In your opinion, which of these people/entities has the most influence on your behavior in combating climate change?^*^ |
|  |  | 4. What drives you to maintain these behaviors daily? Moreover, what might make you give up?^*^ |
|  |  | 5. Where did you learn to carry out these behaviors (school, family, club, media...)?^*^ |
|  |  | 6. With whom do you discuss the importance of these behaviors?^*^ |
|  |  | 7. What led/leads you to perform these behaviors? |
|  |  | 8. What do you think is missing/needed for you to start ... (mention behaviors that participants mention they do not do ‘yet’)? ^*^ |
|  |  | 9. What do you think is the impact of your behaviors on climate change, low, medium, or high? Please, justify.^*^ |
|  |  | 10. What other actions can you take to combat climate change with the help of other people (family, friends, teachers - joint actions and/or influence)? When it does not depend only on you, what do you do to continue combat CC?^*^ |
|  |  | 11. What are the main barriers to adopting actions or behaviors to combat climate change?^*^ |
|  |  | 12. What could help you fight climate change more effectively?^*^ |

*Note*. ^*^Questions asked during the focus group discussion.

## 1.3 Supplementary Table 3. Codebook.

| Category | Category  Definition | Subcategory  Definition | Subcategory  Definition | Example |
| --- | --- | --- | --- | --- |
| Climate  Agency Modes | Individual  Participants’ reports reflect how they “bring their influence to bear on their functioning and environmental events” (Bandura, 2006, p. 165) | Forethought  Participants report how they motivate and guide themselves by creating action plans, adopting goals, and visualizing the likely outcomes of their actions, bringing their influence to bear on their functioning and environmental events (Bandura, 2006, p. 165; Bandura, 2018, p. 131). |  | “It’s the quantity [of plastic bags] that really impresses me. So, it’s something we do right here at home [reusing bags]. When we go shopping, we always keep bags in the car’s boot; this way, nobody forgets. Whenever we go to the supermarket, we just grab a bag, and it is there.” (27ER) |
|  |  | Self-reactiveness.  Participants report how they self-regulate their actions, i.e., through developing appropriate courses of action and motivating and regulating their execution (Bandura, 2006, p. 165; Bandura, 2018, p. 131). |  | “At home, we recycle. I’m always careful when I go out, ah, I think it’s something that I’ve been taught as I’ve grown up, that when I go out, or into nature, I’m always particularly careful not to leave waste on the ground, … be always careful with nature, because it’s something that’s here [around us], right? We have to be very careful, ah...” (4ER) |
|  |  | Self-reflectiveness  Participants report how they reflect on their efficacy in achieving given challenges, the soundness of their thoughts and actions, their values, and the meaning and morality of their pursuits (Bandura, 2006, p. 165; Bandura, 2018, p. 131). |  | "Unfortunately, I could be more ecological than I am. I have a lot of electrical appliances and televisions at home… I know I could live without them, but I am accustomed to them. Hmm, I agree that I'm not the most ecological person ever " (26ER). |
|  | Collective  Participants’ reports reflect how they “pool their knowledge, skills, and resources, and act in concert to shape their future” (Bandura, 2006, p. 165) | Forethought  Participants report how they motivate and guide themselves by creating action plans, adopting goals, and visualizing the likely outcomes of their actions in a group (Bandura, 2006, p. 165; Bandura, 2018, p. 131). |  | “I also hope that schools will soon be able to adopt sustainable energy. I would like to take some initiative to have sustainable energy in the school, and I would like the students and the school to help [set initiatives]. I think we could try to suggest [a list of activities] to the students’ association (…) organize sales and projects to raise money for the school that could help install, at least, a pavilion or a room, or a group of rooms with sustainable energy, or a power station or something like that" (26ER). |
|  |  | Self-reactiveness.  Participants report how they self-regulate their actions; i.e., how they pool their knowledge, skills, and resources, and act collectively to construct appropriate courses of action and to motivate and regulate their execution (Bandura, 2006, p. 165; Bandura, 2018, p. 131). |  | “Beyond actions of civic involvement, I often take part, and people take part with me in [pro-environmental] actions, for example, cleaning beaches, and reforestation.” (11ER) |
|  |  | Self-reflectiveness  Participants report how they reflect on the efficacy of their group in addressing given challenges, the soundness of their thoughts and actions, values, and the meaning and morality of collective pursuits (Bandura, 2006, p. 165; Bandura, 2018, p. 131). |  | “I have taken part many times [actions, for example, cleaning beaches or reforestation], and I think this is one of the actions that not only have an impact in practice but also helps us to be aware of what those actions are, what the problem is, and what our behavior needs to be…” (11ER) |
|  | Proxy  Individuals do not have total control over what happens in their lives (Bandura, 2006, 2018). Participants’ reports reflect how they “influence others who have the resources, knowledge, and means to act on their behalf to secure the outcomes they desire” (Bandura, 2006, p. 165). | Forethought  Participants report how they motivate and guide themselves by creating action plans, adopting goals, and visualizing the likely outcomes of their actions to influence others who have the resources, knowledge, and means to act on their behalf to secure the outcomes they desire (Bandura, 2006, p. 165; Bandura, 2018, p. 131). |  | “It's a real case. I've been talking to the leading teacher of my class [a teacher who manages the class issues and may have power of influence on the school principal] to help me change the situation at the school bar that sells plastic water bottles, like the kind you can only use twice. Each one of us could ask to fill our reusable bottle.” (51ER) |
|  |  | Self-reactiveness.  Participants report how they self-regulate their actions; i.e., how they construct appropriate courses of action and motivate and regulate their execution to influence others who have the resources, knowledge, and means to act on their behalf to secure the outcomes they desire (Bandura, 2006, p. 165; Bandura, 2018, p. 131). |  | “For example, we don't have recycling bins nearby. That's an obstacle, but I've also sent an e-mail to the City Hall, and they came to look at it, and there weren't any great conditions to put it in.” (40ER) |
|  |  | Self-reflectiveness  Participants report how they reflect on their efficacy in realizing given challenges, the soundness of their thoughts and actions, their values, and the meaning and morality of their pursuits to influence others who have the resources, knowledge, and means to act on their behalf to guarantee the results they want (Bandura, 2006, p. 165; Bandura, 2018, p. 131). |  |  |
| Determinants  /Determinants of Climate Agency Modes | Personal Determinants  Personal or individual determinants include biological, cognitive, affective, and other motivational variables within the individual (Bandura, 1999). Specifically, those include self-efficacy, outcome expectations, goals, and self-evaluation (Bandura, 1989). Noteworthy, motivation is self-regulated through internal feedback mechanisms, and conceptions of action result from knowledge gained through direct and indirect instruction (Bandura, 1989, p. 1181) | CC Knowledge  Participants provide information about CC. *Definition of CC:* long-term shifts in Earth’s weather and climate patterns, such as (1) temperature, (2) sea level, (3) precipitation, (4) primarily driven by increased concentrations of greenhouse gas emissions (5) derived from human activities (Intergovernmental Panel on Climate Change, 2023).  Note: This definition aligns with the content addressed in the Portuguese curriculum at the middle and high school levels. | No basis  Participants are not able to define CC. | “Hmm, various climates represent…. Hmm... various themes (...) the climate has nothing to do with... with human things” (16ER) |
|  |  |  | Beginning  Participants define CC by referring to just one aspect of the definition of CC (e.g., CC refers to variations in long-term weather patterns on Earth, such as temperature). | “Well, I think climate change is the change in the Earth’s normal climate, and [temperature] is increasing seriously over a short period of time (…), increasing spontaneously over a short period of time. That’s how I see it.” (12ER) |
|  |  |  | Developing  Participants define CC by referring to at least two aspects of the definition of CC. | “The abnormal things that happen in nature (...) that I believe… weren’t supposed to happen. (...) Among them is the temperature change. (...) Air quality. (...) In this case, the rise in ocean water and the melting of ice (...)” (6ER) |
|  |  |  | Proficient  Participants provide a complete definition of CC. |  |
|  |  | Perceived lack of power  Participants perceive a lack of decision-making power among young people (Line et al., 2010), which is evident across various sectors of their lives. | Social  Participants share their experiences and beliefs: older people do not listen to them and their ideas and efforts to combat CC are frequently ignored. | “At a societal level... hmm, yes, I could try to spread the message of less use [engage in mitigating behaviors]. It’s a bit difficult because right now, I can’t make a big difference in influencing [people], I can’t influence many people in society, don’t you agree?” (26ER) |
|  |  |  | Political  “Young people tend to see governments as having the greatest responsibility for catalyzing a response to CC.” (Corner et al., 2015). However, participants feel that they have no power over the political sector. | “Sometimes I would say a little bit of hope [is needed], because if ordinary persons, can’t do the things themselves to turn everything around, and then you look... at the government not managing things well and … to the many things in society that are put aside, many issues, in favor of the economy, you get a bit discouraged and start thinking: ‘if they [government] don’t make efforts ... what difference am I going to make [with my behaviors]’?” (14ER) |
|  |  | Low outcome expectations  Participants’ reports reflect a judgment that the likely consequence of their behavior will be low impact (Bandura, 1986). |  | “But as a normal citizen, the things I’m going to do, for example, on my own, aren’t going to have a very big impact (33ER)  **Counterexample:** “I think my behaviors are already effective.” (50ER) |
|  |  | Self-efficacy  Participants’ reports reflect “judgments of their capabilities to organize and execute courses of action required to attain designated types of performances” (Bandura, 1986, p. 391). Importantly, it is concerned not with the participants’ skills but their judgments of what they can do with their skills” (Bandura, 1986). |  | "I am not able to solve the problem, it’s obvious, but I can do a lot of things to combat CC. I can do my part." (32ER)  **Counterexample:** “if I see someone in the street, or any friend throwing garbage on the ground, I either tell them to pick it up, or I pick it up myself, but I don't think I have the capacity to do anything else.” (21ER) |
|  |  | Concern for the environment/planet  Participants state that what leads them to carry out or maintain behaviors to combat CC is their concern for the environment/planet, or the future of generations. |  | “I think it’s more of a concern, ah, that in the future, okay, we will live in, not me, but for example, my children, or the people who come after me will live in a world that is more destroyed by warming, with worse living conditions than we have in terms of nature, and so on” (8ER) |
|  | Behavioral Determinants  “Behavior is interacting and exerting influence at the meeting and is active throughout events, playing an important role in the production of events,” (Bandura, 1986, p.23), including “choices people make, how they perform, and how they manage their lives.” (Usher & Ford, 2022) | CC mitigation behaviors  Participants provide examples of their CC mitigation behaviors. Mitigation’ focuses on actions “to reduce greenhouse gas (GHG) concentrations through measures that cut GHG emissions or move carbon out of the atmosphere (Anderson, 2012, p. 192). | Energy Systems  This sector embraces a reduction of greenhouse gas emissions through energy saving (e.g., shifting to renewable energy and using less energy). Participants mention using the energy consciously, such as keeping chargers or appliances unplugged when not in use, keeping lights switched off (United Nations Environment Programme, 2024). | "Try not to use so much energy at home, for example, don’t leave all the lights on. (...) Check... when we buy electrical appliances, there are those power guides, I don’t know what they’re called, but they come in those colors and tell you which uses less, and which uses more [energy] (...). Buy the ones that use less." (26ER) |
|  |  |  | Agriculture, Forestry, and Other Land Uses  This sector includes agriculture, forestry, and other land-based industries, through actions such as ecosystem degradation and restoring ecosystems. It is also associated with reducing food waste and switching to more sustainable diets. Participants mention behaviors such as buying local produce, switching to a vegetable-based diet, composting, and participating in habitat conservation or restoration events (United Nations Environment Programme, 2024) | “This is more about food waste … I don’t know if it applies because we buy the food, and half of it goes to waste. I also live in the countryside, so this is my thing. What we do is: we put all the leftovers, all together in a bucket, and then we feed the chickens with them; I don’t know if this applies.” (5ER) |
|  |  |  | Industry  Industry plays an important role in reducing greenhouse gas emissions throughout the life cycle of products, from the extraction of raw materials, through production, use, and disposal. This sector considers the impact of purchases on the planet and the importance of buying products that can be used for as long as possible before being remanufactured or recycled. Participants mention behaviors such as reusing materials, recycling, and taking care of the amount of water used to produce what they buy or consume (United Nations Environment Programme, 2024). | “Paying attention to (…) reducing the containers of food we buy, yes, food. Because every food we buy comes in containers, so we could try to buy on a larger scale [to avoid buying more containers] or quit buying plastic and paper bags.” (5ER) |
|  |  |  | Transport  This sector is associated with using electric vehicles, public transport, bicycles, other non-motorized means of transport, and walking. Participants report prioritizing walking or using public transport, showing a preference for rail transport compared to air transport (United Nations Environment Programme, 2024). | “I, for example, always take public transport, I rarely take private transport, and, for short distances, I often choose to walk instead of taking the bus, for short distances (…)” (11ER) |
|  |  |  | Buildings  This sector is associated with reducing emissions through energy use and choosing more sustainable options for the management of buildings/houses. Participants mention using natural light or solar panels, reusing water, or reducing shower time (United Nations Environment Programme, 2024). | “During the weekend, when I'm at home, in my room, instead of having the lights on and the air-conditioning on in the cold, I prefer to open the windows, I have sunlight, which I really love, and which I think is an incredible source of light. And I use it a lot, and I only really switch on the light, even if it's necessary” (50ER).  “I always turn off the tap when I'm washing the dishes, etc. Even the flush, I don't waste all the water, I just leave a little...” (40ER). |
|  |  | Lack of control  Participants report difficulties maintaining actions to combat CC, due to, for example, sloppiness, forgetfulness, or preference for immediate comfort. |  | “I think that sometimes it’s sloppiness on my part. Sometimes I want to know, and I care [CC topics], but there are times when I think, ‘Ah, it’s not for me, don’t worry’. I think I must be more careful [with the CC topic].” (4ER)  “Seeing that I’m sitting at home doing nothing and that I could be doing something to help the environment (…) maybe it’s lack of will, the lack of will, it hurts to say, that prevents doing more, even in the situations in which we could help.” (9ER) |
|  |  | Searching for information  Participants report that when they are interested or have questions about CC, they try to find out more by researching or talking to an adult. |  | “(…) videos we also see on the Internet talking about how the planet is doing (…) Sometimes they come up, and I’m interested, and I research more, but I usually research when I have doubts about something.” (29ER)  **Counterexample:** “We talk about it here at home, but I think we could talk about it more. Ah..., I could too, I could do more research and look at other ways to improve.” (4ER) |
|  |  | Inform or influence others about CC  Participants refer to the need to inform, sensitize, or influence others about CC to help combat it. | Family  Participants mention informing, sensitizing, or influencing the family about CC to help combat it. | “For example, my grandparents also use plastic bags; they always reuse them, also because of our influence, because we have told them that it is good.” (24ER) |
|  |  |  | School  Participants mention informing, sensitizing, or influencing their school about CC to help combat it. | “For example, if we see a light that is not necessary at school, we have the habit of asking the school management to turn it off. My school is a very old building and there is a lot of light in hallways that are not even used... So, whenever we pass by (my classmates and I) and see a light on, when it is not needed, we let them know.” (40ER) |
|  |  |  | Friends  Participants mention informing, sensitizing, or influencing their friends about CC to help combat it. | “I try my best to inform my family, my friends, my colleagues that we have to take these measures into account, because... then, planet is dying!” (14ER) |
|  |  | Collective Actions  Participants report carrying out group actions in favor of the environment to combat CC (e.g., collecting trash from beaches with friends) |  | “Besides civic involvement actions, I often take part, and people take part with me, in actions such as beach clean-ups” (11ER) |
|  | Environmental Determinants  Environmental determinants include one's physical and social surroundings, peer groups, and family, which influence human functioning (Usher & Ford, 2022). | Family  Participants mention how their family (e.g., parents) influences their thoughts, behaviors, and feelings through modeling, tuition, or social persuasion” (Bandura, 1986). |  | "I’d say it was my family first, because they ingrained these behaviors in me at a very young age." (14ER) |
|  |  | School  Participants mention how the school context (e.g., conversations with teachers, educational activities organized by their school) influences their thoughts, behaviors, and feelings through modeling, tuition, or social persuasion. (Bandura, 1986) |  | “At the beach. My teacher told me this last summer, and I tried to follow it. Whenever I went to the beach, I tried to leave with one more thing, and that one more thing was beach waste. So, I always brought beach waste to throw away at home. She did this and asked us to do it too, and we did it.” (9ER)  **Counterexample:** “[One of the main obstacles to adopting behaviors to combat CC] is the lack of information at school, that is, it is not a subject that is discussed that much at school, so I am not very informed about it.” (12ER) |
|  |  | Friends  Participants mention how their friends influence their thoughts, behaviors, and feelings through modeling, tuition, or social persuasion. (Bandura, 1986) |  | "My friends, one hundred percent [influence my mitigation behaviors] (...). If a colleague nudges and says:” Look, try doing it this way”, maybe I’ll be more inclined to do it, not so much by the nudge, but because of seeing him doing it. This way I’m more likely to get into the habit [doing mitigation behaviors]" (22ER).  **Counterexample:** “For example, many people of my age throw trash on the ground and think it’s cool (...). When someone doesn't have this kind of behavior, they start to make fun of them ("for behaving correctly") and it ends up being embarrassing.” (19ER) |
|  |  | Media  Participants mention how media (e.g., television and social media posts) influence their thoughts, behaviors, and feelings through modeling, tuition, or social persuasion (Bandura, 1986). |  | "I would say that the improvements in my general knowledge come from the Internet, particularly from these two movements, Team Sme's and Team Triz. Although I have a good general picture of things… Ah, the numbers they show and their approach to the situation [CC] explain very well the problem itself [CC], and the urgency of a [CC] revolution. (22ER)  **Counterexample:** “I think it's a lot about the media, about TikTok. Sometimes you go to TikTok and you see those girls doing the hauls, like “oh, today I have, I bought something at Zara [cloths store]”, and I think, oh, I want it too, I'm going to buy it at Zara. In my opinion, I also think we're very influenced [negatively].” (4ER) |
|  |  | Lack of community participation  Participants complain about the lack of community participation. They mention the need for more people around them to be involved in the fight against CC so that they feel motivated to adopt more environmentally friendly behaviors. |  | “And so it is also a bit about having more people to do things when it comes to not so much personal actions, but in the case of cleaning beaches, planting trees, those two are the main ones” (22ER). |
|  |  | Lack of resources  Participants report a lack of economic and material resources (e.g., access to renewable energy, recycling bins, or infrastructure) as an obstacle to carrying out more behaviors to combat CC. |  | “I think people could make an effort to, it does not have to be all the time, but at least sometimes (…) try to use public transport (...). Unfortunately, I do not do it anymore because in the neighborhood where I live, there is almost no [public] transport; I also think they [city council] could set more [public] transport. Cities could focus a bit more on that.” (5ER)  “It is time, hmm, to really find more affordable ways to combat climate change. I understand that, for the time being, sustainable energies are difficult to obtain (...) [due to] economic and social conditions in general.” (26ER)  **Counterexample:** “Nowadays, we already have recycling bags at school. Schools are already talking about reusable bottles; supermarkets are selling them more easily; I think it is really the people saying I want to do this; I want to be able to (...) help nature and not pollute.” (4ER). |
| References  Anderson A. (2012). Climate change education for mitigation and adaptation. *J. Educ. Sustain. Dev.* 6, 191–206. doi: 10.1177/0973408212475199  Bandura, A. (1986). *Social Foundations of Thought and Action: a Social Cognitive Theory*. Englewood Cliffs, NJ: Prentice-Hall.  Bandura, A. (1989). Human agency in social cognitive theory. *Am. Psychol.* 44, 1175–1184. doi: 10.1037/0003-066X.44.9.1175  Bandura, A. (1999). Social cognitive theory: an agentic perspective. *Asian J. Soc. Psychol.* 2, 21–41. doi: 10.1111/1467-839X.00024  Bandura, A. (2006). Toward a psychology of human agency. *Perspect. Psychol. Sci.* 1, 164–180. doi: 10.1111/j.1745-6916.2006.00011.x  Bandura, A. (2018). Toward a psychology of human agency: pathways and reflections. *Perspect. Psychol. Sci.* 13, 130–136. doi: 10.1177/1745691617699280  Corner, A., Roberts, O., Chiari, S., Völler, S., Mayrhuber, E. S., Mandl, S., et al. (2015). How do young people engage with climate change? The role of knowledge, values, mes­sage framing, and trusted communicators. *Wiley Interdiscip. Rev. Clim. Chang.* 6, 523–534. doi: 10.1002/wcc.353  Intergovernmental Panel on Climate Change (2023). *Climate Change 2023: Synthesis Report. Contribution of Working Groups I, II and III to the Sixth Assessment Report of the Intergovernmental Panel on Climate Change*. [Edited by H. Lee and J. Romero]. Geneva, Switzerland.  Line, T., Chatterjee, K., and Lyons, G. (2010). The travel behaviour intentions of young people in the context of climate change. J. Transp. Geogr. 18, 238–246. doi: 10.1016/j.jtrangeo.2009.05.001  United Nations Environment Programme (2024) The sectoral solution to climate change. Available online at: https://www.unep.org/interactive/sectoral-solution-climate-change/  Usher, E., and Ford, C. J. (2022). *Social Cognitive Theory and Personal Agency*. London: Routledge. | | | | |

## 1.2 Supplementary Table 4. Findings by school level.

| Category | Category | Subcategory | Subcategory | Middle School  *n* (%) | High School  *n* (%) |
| --- | --- | --- | --- | --- | --- |
| Modes of agency | Individual | Forethought |  | 7 (47%) | 4 (24%) |
|  |  | Self-reactiveness |  | 12 (80%) | 13 (77%) |
|  |  | Self-reflectiveness |  | 4 (27%) | 6 (35%) |
|  | Collective | Forethought |  | 6 (40%) | 3 (18%) |
|  |  | Self-reactiveness |  | 7 (47%) | 5 (29%) |
|  |  | Self-reflectiveness |  | 0 (0%) | 1 (6%) |
|  | Proxy | Forethought |  | 1 (7%) | 0 (0%) |
|  |  | Self-reactiveness |  | 1 (7%) | 1 (6%) |
|  |  | Self-reflectiveness |  | 0 (0%) | 0 (0%) |
| Determinants of Human Modes of Agency | Personal Determinants | CC knowledge levels | No basis | 11 (42%) | 8 (32%) |
|  |  |  | Beginning | 5 (19%) | 4 (16%) |
|  |  |  | Developing | 10 (39%) | 13 (52%) |
|  |  |  | Proficient | 0 (0%) | 0 (0%) |
|  |  | Perceived lack of power | Social | 4 (27%) | 5 (29%) |
|  |  |  | Political | 2 (13%) | 4 (24%) |
|  |  | Low outcome expectations  Counterexample |  | 14 (93%)  3 (20%) | 15 (88%)  4 (24%) |
|  |  | Self-efficacy  Counterexample |  | 7 (47%)  6 (40%) | 9 (53%)  7 (41%) |
|  |  | Concern for the environment/planet |  | 12 (80%) | 13 (76%) |
|  | Behavioral Determinants | CC mitigation behaviors | Energy Systems | 6 (40%) | 5 (29%) |
|  |  |  | AFOLU | 7 (47%) | 7 (41%) |
|  |  |  | Industry | 14 (93%) | 16 (94%) |
|  |  |  | Transport | 8 (53%) | 11 (65%) |
|  |  |  | Buildings | 8 (53%) | 6 (35%) |
|  |  | Lack of control |  | 7 (47%) | 13 (76%) |
|  |  | Searching for information  Counterexample |  | 7 (47%)  3 (20%) | 6 (35%)  6 (35%) |
|  |  | Inform or influence others about CC | Family | 4 (27%) | 3 (18%) |
|  |  |  | School | 0 (0%) | 2 (12%) |
|  |  |  | Friends | 2 (13%) | 5 (29%) |
|  |  | Collective actions |  | 7 (47%) | 5 (29%) |
|  | Environmental Determinants | Family |  | 15 (100%) | 15 (88%) |
|  |  | School  Counterexample |  | 15 (100%)  5 (33%) | 16 (94%)  8 (47%) |
|  |  | Friends  Counterexample |  | 2 (13%)  1 (7%) | 7 (41%)  3 (18%) |
|  |  | Media  Counterexample |  | 9 (60%)  0 (0%) | 12 (71%)  2 (12%) |
|  |  | Lack of community participation |  | 8 (53%) | 14 (82%) |
|  |  | Lack of resources  Counterexample |  | 6 (40%)  2 (13%) | 12 (71%)  3 (18%) |

*Note*. Percentages for (sub)categories were calculated based on the number of participants in each part of the interview. First part (i.e., CC knowledge levels): 26 middle and 25 high schoolers participated. Second part: 15 middle schoolers and 17 high schoolers participated. AFOLU: Agriculture, Forestry, and Other Land Uses.

## 2. Supplementary Figure

**Supplementary Figure 1.** Visual tool used to facilitate interviews.
